# Social, Organizational, and Technological Factors Impacting Clinicians’ Adoption of Mobile Health Tools: Systematic Literature Review

**DOI:** 10.2196/15935

**Published:** 2020-02-20

**Authors:** Christine Jacob, Antonio Sanchez-Vazquez, Chris Ivory

**Affiliations:** 1 Anglia Ruskin University Cambridge United Kingdom; 2 University of Applied Sciences Northwestern Switzerland Brugg Switzerland; 3 Innovation and Management Practice Research Centre, Anglia Ruskin University Cambridge United Kingdom

**Keywords:** telemedicine, smartphone, cell or mobile phone, electronic health record, workflow, workload, workplace, public health practice, technology, perception, health education, mHealth, mobile health, telehealth, eHealth

## Abstract

**Background:**

There is a growing body of evidence highlighting the potential of mobile health (mHealth) in reducing health care costs, enhancing access, and improving the quality of patient care. However, user acceptance and adoption are key prerequisites to harness this potential; hence, a deeper understanding of the factors impacting this adoption is crucial for its success.

**Objective:**

The aim of this review was to systematically explore relevant published literature to synthesize the current understanding of the factors impacting clinicians’ adoption of mHealth tools, not only from a technological perspective but also from social and organizational perspectives.

**Methods:**

A structured search was carried out of MEDLINE, PubMed, the Cochrane Library, and the SAGE database for studies published between January 2008 and July 2018 in the English language, yielding 4993 results, of which 171 met the inclusion criteria. The Preferred Reporting Items for Systematic Review and Meta-Analysis guidelines and the Cochrane handbook were followed to ensure a systematic process.

**Results:**

The technological factors impacting clinicians’ adoption of mHealth tools were categorized into eight key themes: usefulness, ease of use, design, compatibility, technical issues, content, personalization, and convenience, which were in turn divided into 14 subthemes altogether. Social and organizational factors were much more prevalent and were categorized into eight key themes: workflow related, patient related, policy and regulations, culture or attitude or social influence, monetary factors, evidence base, awareness, and user engagement. These were divided into 41 subthemes, highlighting the importance of considering these factors when addressing potential barriers to mHealth adoption and how to overcome them.

**Conclusions:**

The study results can help inform mHealth providers and policymakers regarding the key factors impacting mHealth adoption, guiding them into making educated decisions to foster this adoption and harness the potential benefits.

## Introduction

Mobile health (mHealth) is one of the key areas of medical technology innovation that hold promise for reduction of cost, enhancement of health care access, and improvement in the quality of patient care [1-5]. It is also helping to shift the focus of health care to a more patient-centric model that goes beyond treating disease to a more predictive and preventative approach [6,7].

Although the body of evidence that proves the potential value of mHealth is growing, there are still cases where users, mostly clinicians, resist its adoption [8]. For example, scholars in the area of mHealth adoption, such as Gagnon et al [9], found in a previous systematic review that, in reality, many studies reported that health care professionals perceive factors such as mHealth cost more as a barrier than a facilitator. Furthermore, Brewster et al [10] reported in their systematic review on the same topic that clinicians perceive some negative impacts of mHealth on elements such as their credibility and autonomy, affecting staff acceptance of such tools. This should not be overlooked, given that previous research shows that clinicians’ adoption is one of the most influential factors regarding the success of mHealth tools [11-15]; hence, the need and value of better understanding the factors impacting clinicians’ adoption in this context.

The World Health Organization’s global observatory of electronic health (eHealth) considers mHealth a subcategory of eHealth and defines it as “medical and public health practice supported by mobile devices, such as mobile phones, patient monitoring devices, Personal Digital Assistants (PDAs), and other wireless devices.” Telemedicine is, in turn, a subcategory of mHealth and defined as “the communication or consultation between health professionals about patients using voice, text, data, imaging, or video functions of a mobile device. But it can be applied to other situations; the management of chronic diseases of patients living at home is one example.” [16].

According to the diffusion of innovations theory [17], technology adoption studies should look into not only users’ acceptance or rejection of specific innovations but also to what extent innovation is incorporated into a suitable context. Straub [18] examined the most prevalent technology adoption theories—Rogers's innovation adoption and diffusion theories, the Concerns-Based Adoption Model, the Technology Acceptance Model (TAM), and the unified theory of acceptance and use of technology (UTAUT)—and concluded that the process of user adoption of new technologies is complex, fundamentally social, and progressive. This complexity results from the users’ unique views of technology that impact their decision to adopt or reject new technology, highlighting the importance of considering social and organizational factors to enable the successful adoption of new technological tools.

We were guided in our thinking about technology adoption by the field of social studies of technology; we view technology, roles, and practices and organizational structures as interacting parts of a mutually constituting ensemble of elements [19-22]. It follows that it is not simply a matter of *factors* affecting the decision to adopt a technology or not but also of the use of technologies enabling and triggering new forms of organizing and new work practices [23,24]. A *mutually constituting* sensibility alerts the researcher to the fact that the adoption of technology can be part of a deliberate change process and can result in new practices and different uses and interpretations of the technology itself. We are mindful of how such things as organizational culture and existing roles and practices are implicated not just in the decision to adopt but how the decision to adopt can lead to experimentation with new practices and organizational forms. The scope of our literature review engages in more depth with these broader concerns and interests than previous reviews.

A systematic review of relevant literature was carried out to provide an accurate and up to date account of factors that impact clinicians’ adoption of mHealth tools both from a technology and a social and organizational perspective. This work complements a larger ongoing research project and supplements its initial findings, which have already been published [25].

In light of Leonardi’s *Methodological Guidelines for the Study of Materiality and Affordances* [22], the authors analyzed the included studies following three key steps: (1) identifying utility and limitations of the studied solutions, (2) recognizing the real constraints upon opportunities faced by clinicians when using them, and (3) understanding the workflow advantages and disadvantages related to them, as reported in the included articles.

Findings from this review should benefit mHealth providers and policymakers by presenting them with an up to date and comprehensive review of key factors impacting clinicians’ adoption of mHealth tools, as reported in the academic literature. This can inform and guide them in the development of a strategy for promoting the adoption of these tools and enable them to realize their potential benefits.

## Methods

### Overview

The methods for this review were drawn from the Preferred Reporting Items for Systematic Review and Meta-Analysis (PRISMA) guidelines [26]and the Cochrane Handbook [27], both of which provide guidance toward a rigorous and reliable literature review methodology. The review methods were defined in advance and the protocol was published in the PROSPERO (International Prospective Register of Systematic Reviews) and is available on their website [28]. The analysis did not necessitate any major divergence from the initial protocol.

The key question that guided this review was the following: “According to the literature, what are the social, organizational and technological factors impacting clinicians' adoption of mHealth tools?”

### Search Strategy

A search of MEDLINE, PubMed, the Cochrane Library, and the SAGE database in August and September 2018 identified the relevant studies. The scope was narrowed to studies published in the English language between January 2008 and August 2018. Only original, peer-reviewed, and published papers were included. Other forms, such as editorials, unsystematic reviews, interviews, comments, unstructured observations, and position papers, were excluded.

It was decided not to include articles on the basis of on hand searches of reference lists for causes summarized in the Cochrane Handbook: “positive studies are more likely to be cited” and “retrieving literature by scanning reference lists may thus produce a biased sample of studies” [27].

**Figure 1:**
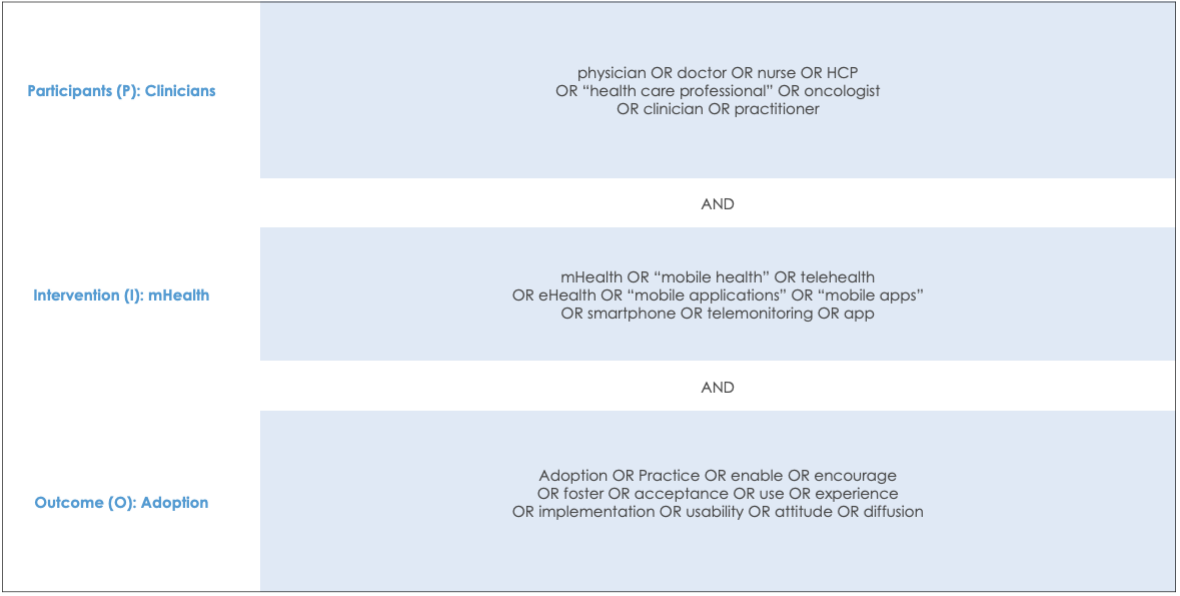
The search string according to the participants, intervention, comparator, and outcome (PICO) framework. mHealth: mobile health.

The search string shown in Figure 1 was developed according to the participants, intervention, comparators, and outcome (PICO) framework [29]. There were no limitations on the kinds of conditions qualified for inclusion, and both qualitative and quantitative studies were included. Comparators were not applicable to this study. Participants (Clinicians) included studies focusing on clinicians and health care professionals. For the review, the Merriam-Webster dictionary’s definition of the word *clinician* was used: “a person qualified in the clinical practice of medicine, psychiatry, or psychology as distinguished from one specializing in laboratory or research techniques or in theory” [30]. Interventions (mHealth) included studies involving smart device use, such as mHealth apps or telehealth. Outcomes (Adoption) included studies addressing the factors impacting mHealth technology adoption or use.

### Study Selection

Independent researchers, CJ and ASV, were involved in the screening, eligibility, and inclusion phases, and any divergences were agreed in discussion between the 2. In the cases where they could not reach an agreement, a third reviewer, CI, discussed it with them and took the final decision. The research team used the open-source app Rayyan QCRI (Qatar Computing Research Institute) to facilitate collaborative screening by the team [31]. Screening lasted from August 2018 to February 2019. A screenshot of the app is included in Multimedia Appendix 1.

The inclusion and exclusion criteria, detailed in Textbox 1, were also developed according to the PICO framework. Studies were excluded if they did not involve the use of mHealth or smart devices; focused solely on, for example, patients, caregivers or technology providers, not including clinicians; were not peer-reviewed; were editorials, interviews, comments, unstructured observations, or position papers; did not address the factors impacting adoption; or if the full text was not available, freely available, or available in English.

After completing the screening, and resolving any conflicting views between the researchers, the selected full texts were assessed for eligibility by CJ and AV, independently. Any disagreements were resolved by discussing with CI.

Subsequently, the risk of bias was assessed using the Critical Appraisal Skills Program tool [32]. The checklist is included in Multimedia Appendix 2, and an Excel sheet with the appraisal of the included studies can be accessed in Multimedia Appendix 3. On the basis of the appraisal, 38 out of the 171 studies did not report a clear participant recruitment strategy, 40 papers did not give enough details on their data collection techniques, 76 did not clarify how they addressed potential ethical considerations, and 25 were not clear enough about their data analysis strategy and whether it was sufficiently rigorous. Articles were not excluded on the basis of technical quality to enable the researchers to capture both theoretical and empirical contributions from the published studies.

**Inclusion and exclusion criteria according to the participants, intervention, comparator, and outcome (PICO) framework.**

**Population (P)**

- Include: Focused on health care professionals (eg, physicians and nurses).
- Exclude: Focused only on patients, caregivers, or technology providers.

**Intervention (I)**

- Include: Focused on solutions involving a smart device (eg, mHealth apps and telehealth).
- Exclude: Using other technologies (eg, virtual reality and machine learning).

**Comparators (C)**

- Does not apply.

**Outcome (O)**

- Include: Addresses factors impacting clinicians’ adoption, acceptance, use, experience, implementation, usability, or attitude of using mHealth for health care service delivery, regardless of the condition.
- Exclude: Focused only on mHealth success or development in general.

**Publication type**

- Include: Original, peer-reviewed, and published paper.
- Exclude: Editorials, interviews, comments, unstructured observations, and position papers, or similar publications.

### Data Collection and Synthesis

The variety of measures and outcomes that were identified in the included articles were not homogenous enough to enable a quantitative synthesis of the data. Therefore, a narrative synthesis was used and structured around the organizational and technological factors impacting clinicians’ adoption of mHealth solutions. QSR NVivo, a computer-assisted qualitative data analysis software, was used to assist in this task.

Data coding began with an initial data extraction grid that included themes based on previous research and technology acceptance frameworks; more themes were added as they emerged during the review process. Braun and Clarke’s thematic analysis [33] was used to identify and extract themes that addressed the review’s research question. The phases of the thematic analysis are explained in detail in Multimedia Appendix 4.

The research themes were also guided by Leonardi’s *Methodological Guidelines for the Study of Materiality and Affordances* [22]; hence, they were split into two key groups, on the one hand, the technological factors, and on the other the social and organizational factors. There was an additional category for implications for social and organizational practices. This process lasted from February to July 2019.

## Results

### Overview

**Figure 2:**
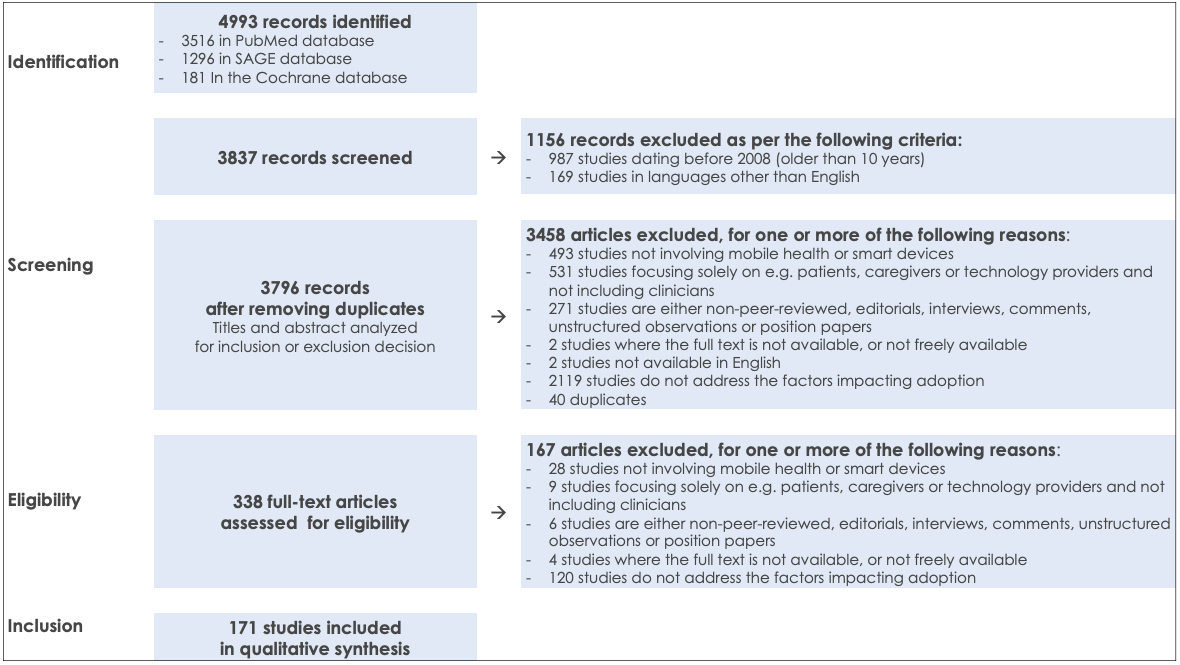
Study selection flow diagram on the basis of the Preferred Reporting Items for Systematic Review and Meta-Analysis (PRISMA) guidelines.

As shown in the study selection flow diagram, visualized in Figure 2, the search string yielded a total of 4993 studies, out of which 3516 from PubMed, 1296 from SAGE, and 181 from the Cochrane database. From these, 1156 studies were excluded after limiting the scope to studies published in English and published after January 2008, leaving 3837 studies for screening. Screening of the titles and abstracts excluded another 3458 articles because 493 of them did not involve mHealth or smart devices, 531 focused solely on nonclinician populations such as patients, caregivers, or technology providers, 271 were editorials, interviews, comments, unstructured observations, position, or non–peer-reviewed papers, 2 were not available as full text, 2 were not available in English, 2119 did not address factors impacting adoption, and 40 were duplicates.

In the eligibility phase, 338 articles were included for full-text assessment. In total 167 articles were excluded for the following reasons: 28 for not involving mHealth or smart devices; 9 for focusing solely on nonclinician populations such as patients, caregivers, or technology providers; 6 for being either, editorials, interviews, comments, unstructured observations, position, or non–peer-reviewed papers; 4 because the full text was not available; and 120 for not addressing the factors impacting adoption. This resulted in the inclusion of 171 studies in the qualitative synthesis.

### Characteristics of Included Studies

The sample characteristics of the included articles are detailed in Table 1. Overall, 62 studies focused on clinicians, 41 on physicians, 21 on nurses, and 46 included clinicians and other populations such as patients or caregivers. From a specialization perspective, some were more represented than others in the included studies; 17 studies focused on primary and acute care, 12 on chronic obstructive pulmonary disease, or congestive heart failure, or cardiovascular disease, 10 on diabetes, 9 on general and family practices, 8 on psychology and mental health, whereas the other specialties were represented four times or less in the included studies.

The majority of the publications did not mention the use of a theoretical framework. Among those that used one, the TAM was the most common (n=19), followed by the theory of diffusion of innovation (n=11), and the UTAUT (n=6). Other models were used once or twice, as detailed in Table 1. From a geographical perspective, 38 studies were conducted in the United States, 22 in the United Kingdom, 15 in Australia, 9 in Canada, 7 in Germany, 7 in Spain, whereas other geographies were covered in 4 studies or less.

Finally, 31 studies were identified as pilot projects. Such studies can be particularly relevant to mHealth providers when rolling out a new tool, as they provide insights into the potential *teething problems* that they can avoid to have better chances for success.

**Table 1:** Characteristics of included studies.

Study characteristic		References
**Study Design**		
	Qualitative (n=64)	[1,2,4,34-94]
	Quantitative (n=58)	[3,95-150]
	Mixed methods (n=32)	[151-181]
	Systematic review (n=11)	[5,9,10,182-189]
	Others (n=5)	[8,190-193]
**Sample size**		
	Less than 10 (n=8)	[67,71,76,85,88,93,171]
	10-20 (n=41)	[1,2,4,10,34,37,39,40,42,46,49,50,54,56,57,61,63-66,68,72,75,79,80,82,86,89-92,141,150,154,157,163, 180,181,183,193,194]
	21-40 (n=30)	[9,35,36,41,44,45,47,51,53,55,60,62,69,70,73,74,77,78,81,95,101,110,131,135,155,165,166,172,176,195]
	41-60 (n=11)	[3,48,52,58,59,84,119,153,156,175,192]
	61-80 (n=8)	[38,87,114,115,137,174,191,196]
	81-100 (n=5)	[97,132,146,160,179]
	More than 100 (n=61)	[8,43,83,96,98-100,102-109,111-113,116-118,120-130,133,134,136,138-140,142-145,147-149,151,152,158,159, 161,162,164,167-170,173,177,190]
**Sample composition**		
	Clinicians (n=62)	[9,35,36,40,45,47,53,55,56,60-62,64,66,67,69,70,73-75,79,81,82,85,86,90,91,93,97,98,101,104,106, 107,110,112,119,120,126,130,131,137-139,141,145,147,148,154,159,164,168,171,179,180,186,189,191,193,194]
	Clinicians plus others (eg, patients) (n=46)	[2,4,5,8,34,38,41,43,48,51,52,58,59,68,78,83,84,87,92,94,103,115,117,151-153,155-158,160,162,163,166,167,169, 174,176-178,182-184,187,192,195]
	Physicians (n=41)	[1,3,37,39,46,49,50,54,77,89,95,99,100,102,105,108,113,114,116,118,121-123,125,127-129,132-134,136,140,143, 144,149,150,161,165,170,175,190]
	Nurses (n=21)	[10,42,44,63,71,72,76,88,96,109,111,124,135,142,146,172,173,181,185,188,196]
**Specialty or condition**		
	Primary or acute care (n=17)	[1,42,43,48,59,68,72,94,103,130,132,139,169,183,184,187,192]
	Chronic obstructive pulmonary disease, congestive heart failure, and cardiovascular disease (n=12)	[10,41,52,71,78,80,83,97,104,108,119,164]
	Diabetes (n=10)	[38,51,75,88,92,115,125,147,160,162]
	General and family practice (n=9)	[39,49,89,102,117,121,128,134,144]
	Psychology and mental health (n=8)	[2,73,81,90,112,153,166,194]
	Dermatology (n=4)	[1,54,126,152]
	Substance use recovery (n=4)	[61,67,158,167]
	Residential aged care, home health nursing (n=4)	[146,156,171,182]
	Pediatric, maternal (n=4)	[57,74,141,168]
	Neurology, stroke (n=4)	[69,123,150,176]
	Intensive care unit (n=4)	[109,111,186,188]
	Asthma (n=3)	[70,77,84]
	Oncology (n=3)	[53,155,180]
	Sexual health, HIV (n=3)	[138,163,177]
	Others (n=13)	Ambulatory care [149], cognitive behavioral therapy [120], emergency [159], genetics [91], geriatrics [40,60], hypertension [5], nephrology [87], obesity and irritable bowel syndrome [36], otolaryngology [100], radiology [131], speech-language pathology [50], tuberculosis [58]
**Location**		
	The United States (n=38)	[1,2,36,45,53,54,57,60,61,67,68,75,77,88,93,99,102,109-112,120,121,123,127,137,138,141,159,166-169, 173,194-196]
	The United Kingdom (n=22)	[10,41,43,48,49,55,62,70,71,79,80,83,94,115,124,154,163,165,174,176,179,180]
	Australia (n=15)	[39,42,50,51,69,73,81,91,92,103,153,156,161,164,171]
	Canada (n=9)	[3,52,76,78,87,146,155,178,192]
	Germany (n=7)	[100,108,117,134,135,149,150]
	Spain (n=7)	[59,97,122,126,132,139,143]
	Norway (n=4)	[85,86,89,90]
	South Korea (n=4)	[8,107,114,129]
	Sweden (n=4)	[46,72,82,175]
	Austria (n=3)	[98,160,191]
	Iran (n=3)	[131,147,170]
	The Netherlands (n=3)	[84,142,152]
	Taiwan (n=3)	[35,113,116]
	Others (n=39)	Argentina [44], Australia–United Kingdom [65], Austria–Sweden [140], Bangladesh [56], Belgium [162], Brazil [37], Congo [193], Ethiopia [105], Europe [66,119], France [40,128], Ghana [74], Iraq [95], Israel–Portugal [34], Italy [158], Japan [125], Japan–Sweden [104], Jordan–Syria [151], Lebanon [190], Malaysia [145], Nepal [4], the Netherlands–Spain–United Kingdom [38], New Zealand [63,172], Nigeria [106], North America–Europe [130], Poland [96], Portugal [157], Russia [58], Senegal [118], South–North America [148], Spain–Colombia–Bolivia [133], Sri Lanka [47], Switzerland [181], Syria [101], Turkey [136,144], the United States–South Africa–Thailand–Peru [177]
**Theoretical framework**		
	TAM^a^ was the most used theoretical framework (n=19)	[90,97,102,111,126,127,129,132-136,139,146,149,168,170,173,196]
	The theory of diffusion of innovation (n=11)	[47,52,73,115,120,127,133,134,138,175,196]
	UTAUT^b^ (n=6)	[38,98,106,107,142,181]
	Others (n=23)	Affordability, practicability, effectiveness, acceptability, safety or side effects, and equity criteria [39]; Consolidated Framework for Implementation Research [61,86,166]; design science research methodology [157]; Giddens’s concepts from structuration theory and consequence of modernity [79]; organizational readiness for change model [168]; organizational theory of implementation effectiveness [169]; reach, effectiveness, adoption, implementation, and maintenance framework [167]; sociotechnical theory [94,192]; stakeholder empowered adoption model [174]; the technology-organization-environment framework [95]; technological frames [46]; the dual-factor model [116]; the normalization process theory [65]; theory of change [176]; theory of planned behavior [113,132]; theory of reasoned action [132,133]; theory of technology readiness [133]; Updated DeLone and McLean Information System Success Model [125]
**Pilot projects**		
	Studies identified as pilot projects (n=31)	[43,48,53,66,68,74,78,87-89,94,95,98,112,114,126,137,139,158,162-167,171,174,176,181,192,193]

^a^TAM: Technology Acceptance Model.

^b^UTAUT: unified theory of acceptance and use of technology.

### Technological Factors

**Figure 3:**
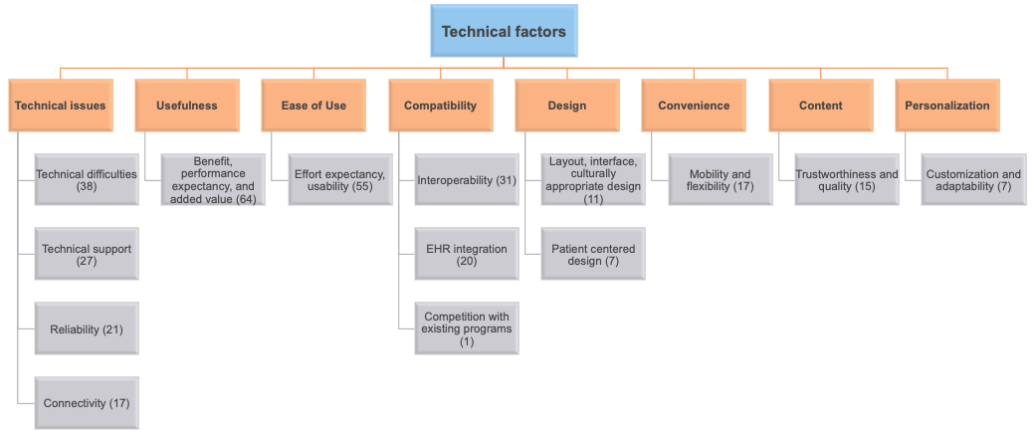
Overview of technological factors and their occurrence. EHR: electronic health record.

The technological factors impacting clinicians’ adoption of mHealth were categorized into 8 key themes: usefulness, ease of use, design, compatibility, technical issues, content, personalization, and convenience. These were, in turn, subdivided into a total of 14 subthemes. Figure 3 gives an overview of these technological factor themes and subthemes and their respective occurrence.

Technical issues were the most prominent factors, often related to matters such as connectivity (n=17), reliability (n=21), technical support (n=27), and technical difficulties in general (n=38). Features determining usefulness, such as expected benefits, performance expectancy, and added value, were also among the most prominent technological factors in the selected studies (n=64). Ease of use, determined by features such as effort expectancy and usability, was also quite central (n=55). Furthermore, several studies raised some concerns related to compatibility, such as interoperability issues (n=31), electronic health record (EHR) integration (n=20), and competition with existing programs (n=1).

Some design-related factors were also cited, such as layout, interface, culturally appropriate design (n=11), and the importance of patient-centered design (n=11). The tools’ convenience, determined by its level of mobility and flexibility, also played a role (n=17), in addition to the trustworthiness and quality of the content (n=15), and personalization possibilities through customization and adaptability (n=7). Table 2 details the technological factors impacting adoption, their occurrence, and the respective studies where they were identified.

**Table 2:** Technological factors and their occurrence, with references.

Factor and subthemes		References
**Technical issues**		
	Technical difficulties (n=38)	[2,9,37,41-44,51,54-56,61,74,75,82,83,86,90-92,101,109,115,128,150,156,162,163, 165,172,177,181,182,186,188,189,194,197]
	Technical support (n=27)	[2,9,10,44,49,51,57,63,68,71,72,79,80,90,92,95,97,98,126,136,139,144,153,154,174, 185,189]
	Reliability (n=21)	[9,10,35,36,44,49,50,55,69,79,91,92,96,108,117,140,152,153,163,177,182]
	Connectivity (n=17)	[37,38,44,50,51,55,68,74,91,95,124,130,153,164,172,181,182]
**Usefulness**		
	Benefit, performance expectancy, and added value (n=64)	[5,9,34,35,38,39,45,47,50,55,60,61,63,64,67,69,78,84-86,89,90,95,98,105-108,111, 112,114-117,122,125-127,129,132,134-136,139,141-147,149,150,153,156,158,163,168,170,172, 173,179,188,189]
**Ease of use**		
	Effort expectancy and usability (n=55)	[4,5,9,10,38,42,44,52,59-61,69,70,72,73,78,81,84,86-89,92,105,106,110-112,116, 120,122,126,129,132,136,141,144,146,149,155,156,158,166,168,170-173,179,181-183, 185,189,192]
**Compatibility**		
	Interoperability (n=31)	[2,9,10,34-36,41,53,54,59,61,66,70,72-74,80,87,95,127,128,134,139,156,165,169,172,174, 184,186,196]
	Electronic health record integration (n=20)	[9,38,48,72,78,84,85,87,129,143,151,162,167,172,174,175,182,188]
	Competition with existing programs (n=1)	[169]
**Design**		
	Layout, interface, and culturally appropriate design (n=11)	[9,38,41,60,73,78,83,155,171,182]
	Patient-centered design (n=7)	[39,77,78,159,162,166,182]
**Convenience**		[5,61,68,73,75,78,82,89,91,116,124,131,136,161,162,165,173]
	Mobility and flexibility (n=17)	
**Content**		[9,38,48,59,66,73,81,114,117,124,144,154,165,175,192]
	Trustworthiness and quality (n=15)	
**Personalization**		[38,70,72,84,124,181,192]
	Customization and adaptability (n=7)	

### Social and Organizational Factors

**Figure 4:**
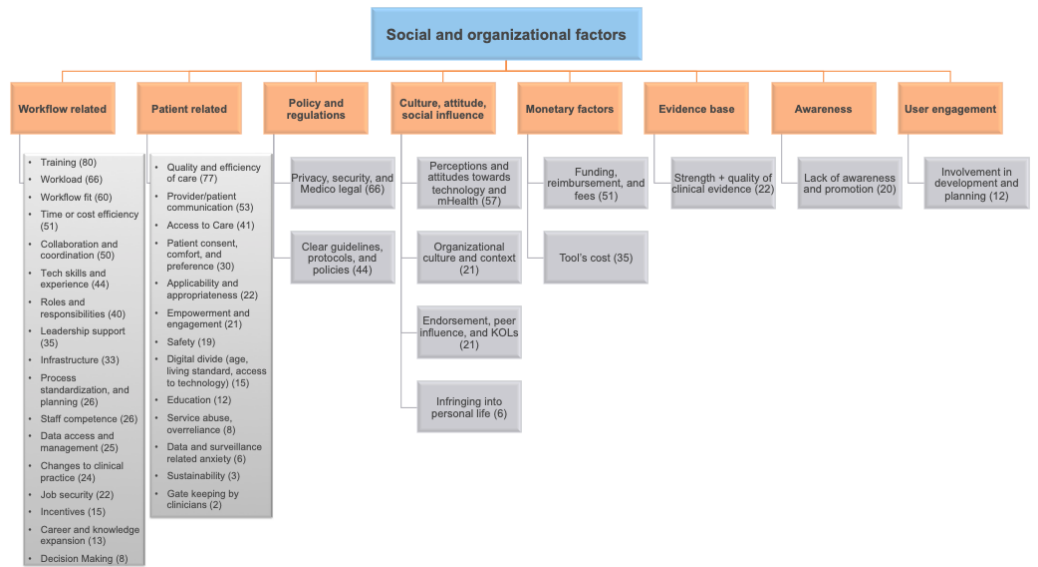
Overview of social and organizational factors and their occurrence. mHealth: mobile health; KOL: key opinion leader.

The social and organizational factors impacting clinicians’ adoption of mHealth were manifestly more numerous than the technical factors. These factors were also categorized into 8 key themes: workflow related, patient related, policy and regulations, culture or attitude or social influence, monetary factors, evidence base, awareness, and user engagement. Key themes were, in turn, divided into a total of 41 subthemes, as shown in Figure 4, which provides an overview of the social and organizational factors and their respective occurrence.

Workflow-related factors were the most prominent organizational factor in the included articles, with 17 subthemes. Training (n=80) was the most central workflow-related theme, followed by workload (n=66), workflow fit (n=60), time and cost efficiencies (n=51), collaboration and coordination (n=50), technical skills and experience (n=44), the impact on roles and responsibilities (n=40), the extent of leadership support (n=35), organizational or local infrastructure (n=33), process standardization and planning (n=26), staff competence (n=26), data access and management (n=25), changes to clinical practice (n=24), job security (n=22), incentives (n=15), impact on career and knowledge expansion (n=13), and decision making (n=8). Table 3 details the subthemes of the workflow-related factors impacting adoption, their occurrence, and the respective studies where they were identified.

**Table 3:** Workflow-related factors and their occurrence, with references.

Factor	Subthemes	References
Training (n=80)	Clinicians’ training to enable an efficient use and management of the tools	[2,4,9,10,37,42-44,49,50,52-56,59,61,62,65,68,70-74,77,79-81, 83,85,89,90,92-94,96,97,103,104,111,112,120,122,126,128,129, 136,139,142,143,149-154,156,158,162,165,166,168-173,176,179,182, 183,185-189,191,194]
Workload (n=66)	Availability and allocation of resources	[2-5,9,10,34-37,39,41,45,46,48,49,51,53,55,59,61,62,70-74,77-81, 83-86,89,90,92,102,104,115,119,122,123,131,152,158-160,162, 164,169,172,174,178,181-183,185,186,188-190,192,195]
Workflow fit (n=60)	Improvements versus disruptions of the workflow and organization of work	[1,8-10,36,39,40,46,48,53,57,59,61,67,70,72-74,78,80,81,83-89,94, 104,107,109,114,120,122,129,135-137,141,147,150,155,156,159, 161,163,166,169,171,172,178,180-182,185,188,193]
Time or cost-efficiency (n=51)	Impact on efficiency and competitiveness	[1,5,9,38,39,43,44,47,49,52-54,57,59-61,64,74,77,78,86,87,91, 94,107-110,114,115,119,132,137,140,150,152,156,157,159,161, 162,165,168,173,174,177,179,181,188,190,195]
Collaboration and coordination (n=50)	Coordination of health services and collaboration between health care professionals	[1,2,8-10,39,40,42,46,50,52,55-57,59,61,68,69,71,84,85,87,88, 92,93,100,102,109,111,127,137,141,150,152,153,155,156,159, 161,162,168,172,176,182,185,186,188,194]
Technical skills, and experience (n=44)	Clinicians’ tech-savviness, and previous experience with technology or mHealth^a^	[9,37,44,49,55,59-61,67,72,73,81,83,96,100-102,112,121,122,127, 129,133,134,136,138,142,143,145,146,149,153,155,157,158,161, 168-170,179,184,188,191,196]
Roles and responsibilities (n=40)	Expansion, reassignment, or possible changes to clinical roles and responsibilities	[2,10,39,40,48,56,59-62,65,67,68,70-72,74,77-80,82,83,85,86, 88-90,94,103,104,152,157,164,167,169,176,182,189]
Leadership support (n=35)	Senior management and organizational support	[5,9,34,35,40,42,59,61,71-73,86,95,103,105,106,111,127,131,132,134, 138,145,156,157,164,166,170,174,181,182,188,192,195,196]
Infrastructure (n=33)	Availability and accessibility of the needed foundation	[2-4,9,35,47,51,54,69,70,77,82,83,87,91,93,95,116,124,126, 127,130,151,153,154,157,163,169,172,179,181,187,193]
Process standardization and planning (n=26)	Governance and control, streamlined procedures, and processes	[5,8,9,39,40,44-46,61,65,67,73,80,82-84,86,88,103,149,155, 160,164,167-169,180,182,185,189]
Staff competence (n=26)	Expertise in the required skills	[2,4,9,36,37,39,51,58,59,69,73,80,84,91,92,105,108,109,137, 154-157,164,176,185]
Data access and management (n=25)	Accessing, analyzing, and interpreting generated data	[36,38,51,53,60,62,66,72-75,77,78,100,108,115,120,150,157, 167,169,176,177,182,183]
Changes to clinical practice (n=24)	New paradigms of care and treatment	[10,35,37,40,46,48,49,59,64,65,72,79,80,92,94,123,130,139, 162,182,185,186,189,192]
Job security (n=22)	Autonomy, loss of control, threat to own career, and professional identity	[9,10,55,62,72,79,80,108,113,116,118,124,130,140,141,151, 159,176,182,185,188]
Incentives (n=15)	Different means to incentivize clinicians	[4,54,59,87,88,106,122,129,141,157,164,168,175,191]
Career and knowledge expansion (n=13)	Impact on professional development and expertise	[62,72-74,86,124,131,137,161,162,169,188,189]
Decision making (n=8)	The process of decision making in a fragmented health care system	[4,8,34,43,71,72,74,97,102,160,191]

^a^mHealth: mobile health.

Patient-related factors arose quite often in the included articles and were split into 13 subthemes. The most prevalent patient-related subtheme was the quality and efficiency of patient care, for example, treatment outcomes, clinical delivery, patient monitoring, and treatment compliance (n=77), followed by the quality and ease of communications between patients and the care team (n=53), enhancing patients’ access to care and reaching the underserved (n=41), then patients’ comfort with technology, personal preferences, and the ease of getting an informed consent from the patients (n=30). Applicability and appropriateness, meaning the suitability of patients on the basis of their needs and characteristics, also occurred quite often (n=24), seeing mHealth as an opportunity to empower and reassure patients and increase their engagement in managing their condition, which was also a key factor (n=21).

In addition to patient safety (n=19), factors included patient age, living standard, and access to technology (n=15), better patient education and awareness (n=12), patient overdependence on practitioner support (n=8), and patients’ worries and anxiety related to the understanding and interpretation of data, or the feeling of being observed (n=6). The least common subthemes were those reflecting concerns regarding patients’ long-term commitment and use (n=3) and protective or paternalistic attitudes of the care team (n=2). Table 4 details the subthemes of the patient-related factors impacting adoption, their occurrence, and the respective studies where they were identified.

**Table 4:** Patient-related factors and their occurrence, with references.

Factor	Subthemes	References
Training (n=80)	Clinicians’ training to enable an efficient use and management of the tools	[2,4,9,10,37,42-44,49,50,52-56,59,61,62,65,68,70-74,77,79-81,83,85,89,90,92-94, 96,97,103,104,111,112,120,122,126,128,129,136,139,142,143,149-154,156,158, 162,165,166,168-173,176,179,182,183,185-189,191,194]
Workload (n=66)	Availability and allocation of resources	[2-5,9,10,34-37,39,41,45,46,48,49,51,53,55,59,61,62,70-74,77-81,83-86,89,90,92, 102,104,115,119,122,123,131,152,158-160,162,164,169,172,174,178,181-183,185,186,188-190, 192,195]
Workflow fit (n=60)	Improvements versus disruptions of the workflow and organization of work	[1,8-10,36,39,40,46,48,53,57,59,61,67,70,72-74,78,80,81,83-89,94,104,107,109, 114,120,122,129,135-137,141,147,150,155,156,159,161,163,166,169,171,172,178,180-182,185, 188,193]
Time or cost-efficiency (n=51)	Impact on efficiency and competitiveness	[1,5,9,38,39,43,44,47,49,52-54,57,59-61,64,74,77,78,86,87,91,94,107-110,114, 115,119,132,137,140,150,152,156,157,159,161,162,165,168,173,174,177,179,181,188,190,195]
Collaboration and coordination (n=50)	Coordination of health services and collaboration between health care professionals	[1,2,8-10,39,40,42,46,50,52,55-57,59,61,68,69,71,84,85,87,88,92,93,100,102,109,111, 127,137,141,150,152,153,155,156,159,161,162,168,172,176,182,185,186,188,194]
Technical skills, and experience (n=44)	Clinicians’ tech-savviness, and previous experience with technology or mHealth^a^	[9,37,44,49,55,59-61,67,72,73,81,83,96,100-102,112,121,122,127,129,133,134,136,138, 142,143,145,146,149,153,155,157,158,161,168-170,179,184,188,191,196]
Roles and responsibilities (n=40)	Expansion, reassignment, or possible changes to clinical roles and responsibilities	[2,10,39,40,48,56,59-62,65,67,68,70-72,74,77-80,82,83,85,86,88-90,94,103,104,152, 157,164,167,169,176,182,189]
Leadership support (n=35)	Senior management and organizational support	[5,9,34,35,40,42,59,61,71-73,86,95,103,105,106,111,127,131,132,134,138,145,156, 157,164,166,170,174,181,182,188,192,195,196]
Infrastructure (n=33)	Availability and accessibility of the needed foundation	[2-4,9,35,47,51,54,69,70,77,82,83,87,91,93,95,116,124,126,127,130,151,153,154, 157,163,169,172,179,181,187,193]
Process standardization and planning (n=26)	Governance and control, streamlined procedures, and processes	[5,8,9,39,40,44-46,61,65,67,73,80,82-84,86,88,103,149,155,160,164,167-169,180, 182,185,189]
Staff competence (n=26)	Expertise in the required skills	[2,4,9,36,37,39,51,58,59,69,73,80,84,91,92,105,108,109,137,154-157,164,176,185]
Data access and management (n=25)	Accessing, analyzing, and interpreting generated data	[36,38,51,53,60,62,66,72-75,77,78,100,108,115,120,150,157,167,169,176,177,182,183]
Changes to clinical practice (n=24)	New paradigms of care and treatment	[10,35,37,40,46,48,49,59,64,65,72,79,80,92,94,123,130,139,162,182,185,186,189,192]
Job security (n=22)	Autonomy, loss of control, threat to own career, and professional identity	[9,10,55,62,72,79,80,108,113,116,118,124,130,140,141,151,159,176,182,185,188]
Incentives (n=15)	Different means to incentivize clinicians	[4,54,59,87,88,106,122,129,141,157,164,168,175,191]
Career and knowledge expansion (n=13)	Impact on professional development and expertise	[62,72-74,86,124,131,137,161,162,169,188,189]
Decision making (n=8)	The process of decision making in a fragmented health care system	[4,8,34,43,71,72,74,97,102,160,191]

Other social and organizational factors included policy and regulations related to privacy or security or medico-legal issues (n=66) and the need for clearer guidelines or protocols or policies (n=44). Cultural and social factors also prevailed quite often and were mostly linked to perceptions and attitudes toward technology and mHealth (n=57), organizational culture and context (n=21), and endorsement or peer influence (n=21).

Monetary factors, such as funding or reimbursement or fees (n=51) and the tools’ cost (n=35), were also central, followed by the strength and quality of clinical evidence (n=22), lack of awareness and promotion (n=20), and user involvement in development and planning (n=12). Table 5 details the other social and organizational factors impacting adoption, their subthemes, occurrence, and the respective studies where they were identified.

**Table 5:** Other social and organizational factors and their occurrence, with references.

Factor and subthemes		References
**Policy and regulations**		
	Privacy, security, and medico-legal issues (n=66)	[2,3,9,10,37-39,44,47,49,52,53,58-61,65,67,73,77,84,90,95,98,100,102,108, 110,114,117,123,125,127,128,130-132,137,138,140,142-144,149,152,156,158,160-163,167, 172,174,176-178,182-185,189-193]
	Clear guidelines, protocols, and policies (n=44)	[1,4,5,9,10,34,35,37,42,43,47,51,61,63,64,67,73,76,92,95,102,104,121-124,130, 141,143,145,151,155-157,159,161,162,168,175,176,178,179,190,193]
**Culture, attitude, and social influence**		
	Perceptions and attitudes toward technology and mHealth^a^ (n=57)	[5,9,10,34,35,37,50,55,58-61,63,64,67,69,83,85,95,97,100,104,107-109,111, 113,116-118,124,125,127,130,132-134,136,138,143,146,147,153,155,157,166,168,173,174, 178,185,186,188,190,191,193,195]
	Organizational culture and context (n=21)	[37,42,45,63,76,86,95,101,113,124,130,145,148,151,161,165,168,170,176,179,193]
	Endorsement, peer influence, and key opinion leaders (n=21)	[2,9,38,51,61,67,99,105,113,125,128,132,142,146,161,163,170,175,181,196]
	Infringing into personal life (n=6)	[47,65,77,161,188,190]
**Monetary factors**		
	Funding, reimbursement, and fees (n=51)	[2-5,8,9,35,36,39,51,54,56,60,61,64,67,68,70,71,74,77,84,86,91,92,100-102, 119,122,123,128-131,141,143,151,152,156,159,160,162,164,167,168,182,184, 186,191,195]
	Tool’s cost (n=35)	[1,2,5,9,34,38,39,41,51,53,55,60,61,63,64,67,73,78,84,91,95,110,114,123,130-132,142, 144,147,156,159,177,182,191]
**Evidence base**		
	Strength and quality of clinical evidence (n=22)	[5,43,55,60,67,69,70,73,84,85,89,108,109,115,155,159,160,162,169,175,186,188]
**Awareness**		
	Lack of awareness and promotion (n=20)	[2,9,38,43,55,56,61,73,89,120,131,138,149-151,156,168,175,180,182]
**User engagement**		
	Involvement in development and planning (n=12)	[10,38,52,61,68,72,86,135,169,172,182,183]

^a^mHealth: mobile health.

## Discussion

The main findings of this review emphasize the principal factors impacting clinicians’ adoption of mHealth tools. Factors’ prevalence sheds light on the clear importance of social and organizational factors that go beyond the technical features, highlighting the importance of taking them into account during the development and deployment of these tools.

### Technological Factors

Technical difficulties were the most cited technical barriers identified in the included articles. Studies reported technical difficulties and limitations in general, besides other issues, such as failing to update the system or testing and installation issues [2,10,51], system errors [74,163], poor output quality (eg, poor images or video quality) [37,82,91,189], login issues [42,86,150,172], and missing functionalities [181]. It was reported that such issues sometimes impacted the recruitment of eligible participants [163], created a feeling of frustration among users [44,75,162], or made the staff more reluctant to promote the tool, as it might not work properly [43], and resulted in interruption of care [75,90,109,188].

Technical support availability and cooperation from the information technology (IT) department can also impact clinicians’ intention to use mHealth tools. Support is, moreover, expected to be available whenever a clinical shift is active such as during the night, weekends, and holidays [44]. The lack of technical support can create difficulties for the adoption, as clinical staff do not want to be expected to do the technical installation and troubleshooting themselves [10]. Some concerns were raised regarding outsourced offshore technical support models that were deemed impersonal and *script-driven* and not very useful [51]. Furthermore, IT departments in hospitals may not be fully prepared for supporting staff’s mobile phones when these are used to access mHealth tools [63]. Although some studies reported staff’s satisfaction with the technical support offered to them, there were still some concerns about delayed delivery of the service because of lack of technical support staff [71,72,79,90].

System reliability can also cause challenges that affect clinicians’ intentions to use mHealth. System failures and malfunctions raised concern for staff [35,140,177], and clinicians who are skeptical about the reliability of the service might refrain from using it altogether [55,108]. Users want to be sure that it will work in every emergency even when patients are using them on their own [69,79]. Similarly, poor signal connectivity linked to speed and quality of the connection can cause major usability issues making the use of such tools almost impossible [37,44,50,95,181], hence diminishing the usefulness of the tool [74] and resulting in frustration and reluctance to use the service [124,153]. An offline functionality whenever possible could help overcome such issue [38], although some clinicians did not perceive slow connections as a significant barrier [68,130]. In some cases, clinical staff developed some work-around routines to overcome connectivity issues by printing the needed patient information, resulting in additional data security and privacy issues [172].

Many of the included studies indicated that usefulness and perceived usefulness have a direct impact on the adoption and intention to use. Clinicians are more likely to use a tool when they understand its benefits [34,78,86], when they find it useful for their daily work [85,89,127,134,146,150,179], and in emergencies [47,188]; they would refrain from using it if they are skeptical about the value it brings to their clinical practice [35,60,69,84,108,115,141,153,156,158], sometimes because of their lack of awareness of studies demonstrating effectiveness [90]. Some studies noted that the positive perception of usefulness usually increases for clinicians that use such tools more frequently [143].

Studies also linked perceived usefulness in many cases to ease of use and effort expectancy. mHealth tools should be very user-friendly and intuitive so that every clinician can use them easily, including those not comfortable with technology [4,70,73,166,189]; otherwise, they might be considered a waste of time [72,156]. Although complex and unintuitive user interfaces are considered a clear barrier [141,155], it was also noted that usability alone is not enough for user acceptance [181]. This was sometimes explained by the specific context that clinicians operate in [168], so ease of use could be perceived as less important in underprivileged contexts where clinicians lack resources and are grateful for any tool that would help facilitate their professional duties [105].

The tools’ technical compatibility also plays a role in its acceptance. Clinicians have a positive attitude toward tools that integrate well with the other systems that they are using on a daily basis [127,196]. Interoperability issues can raise clear concerns when mHealth tools cannot be integrated into the hospital or clinic’s current systems [87,128]. Similarly, the lack of electronic medical records (EMRs) or EHR integration can cause similar issues [9,183]. This can create limitations in data integration and exchange [34,36,156,174] and therefore create duplication of effort and increase workload [70,72,80]. Some clinicians also raised the reliability of self-reported data and the importance of validating it in comparison to the data in the patient’s EMR to make informed decisions [175]. Conversely, a tool that integrates well with current systems would be highly appreciated as it would ensure that patient data is always up to date and would alleviate clinicians’ workload [38,129].

Layout, interface, and culturally appropriate and patient-centered design came up as important influencing factors in several studies. The choice of design and color should be well informed; for example, bright colors should be avoided as they may cause seizures in people suffering from epilepsy [38]. Cluttered and unorganized displays impact adoption negatively [83,155,171]. Users should also be able to adapt design elements, such as font size, according to their own preference [38]. Customization and personalization should also go beyond design elements to include the type of information displayed according to the context and the needs of the individual users [124,181]. Furthermore, clinicians are keen to see tools that can be customized to each patients’ clinical condition [70,84]. Concerns were raised regarding the gap that sometimes exists between the tools’ designers and users, hence the desire for the tools to be adaptable to the user needs, not the other way around [72].

The convenience and mobility of tools accessed via mobile phones were mostly seen as facilitators. They also have a positive impact on perceived usefulness and ease of use [116] and can increase clinicians’ ability to offer care in a timely manner [5,78]. The portability of mHealth tools enabling clinicians to access information and achieve tasks anytime and anywhere was highly valued [73,131,136,161,162]. Conversely, it was also reported that some users perceived the small size of the mobile screen as an inconvenience that could hinder adoption [165].

### Social and Organizational Factors: Workflow Related

Workflow-related factors were the most prominent in the studied articles, with 17 subthemes. Training came up in 80 articles, showing the central role it plays in making or breaking the success of mHealth tools. Several studies identified the impact that appropriate training programs could play in increasing clinicians’ intention to use such tools [9,97,139,149,179].

Factors such as nonexistent, inadequate or insufficient training [4,10,42,72,80,151], lack of time to learn how to use the new tools [81,89,128,183], resources required to ensure the sustainability of training programs [73,156], and training programs that focus solely on the technical side without addressing the workflow changes associated with mHealth use [44,83,94,104,189,194] were among the most important training-related barriers. The significance of training is because of clinicians’ need to develop new skills to be able to benefit from mHealth tools and embed them properly in their work practice [65,70,77,92,122,154]. Proper training also helps achieve the highest potential of such tools, given that research shows that clinicians sometimes do not benefit from all available features simply because they were not aware of them [150].

Factors related to workload and resource allocation were also central. Staff and resources availability and allocation were the main hurdle identified [9,34,51,55,70,85,102,104,152, 181,183,192]. Adequate staffing is considered a prerequisite for successful adoption [2,61,77,78,92,185,189]. Some studies acknowledged that mHealth caused an increase in workload [4,41,195] mostly because of double data entry caused by lack of integration and interoperability [35,72,172], adjustment to new responsibilities and ways of working [48,79,80], and poor workflow adaptability [182]. Moreover, clinicians may refrain from adopting the tools altogether if they believe that they would increase their workload [5,37,174]. This perception is sometimes triggered by their experience with other health IT systems, which added to their workload [46]. At the same time, some studies reported no change in the overall workload because of mHealth adoption [10], whereas others suggested that such technologies can alleviate workload where clinicians’ recruitment and retention are challenging by improving efficiency [73] and providing additional support and resources [45,122,159,185,186].

*Fit* with the clinical practice and compatibility with workflow are also significant requirements for a successful adoption [9,59,84,114,120,136,163,166,181,184]; accordingly, proper planning and integration [8,10,61,73,182] and a good understanding of treatment processes [78,129,172] are essential to avoid any disruption for clinical practice. Clinicians’ perceptions that these technologies might negatively impact their work processes can hinder adoption [46,53,80,141]. Such assessments are sometimes because of poor adaptability of the current routines to mHealth [88,182]. Nonetheless, mHealth adoption can also sometimes result in workflow modification, where an adaptation of working patterns is necessary to harness the potential of such tools [67,94,178]. These changes are mostly aimed at complementing routine care rather than replacing it [70,180], and reorganizing work to warrant routine practice.^39^,^40^,^122^,^150^

mHealth could enhance competitiveness through time and cost efficiencies, optimized work patterns [1,54,59,74,86,109, 152,161,188,190,195], quicker access to care [91,137,159,162], and rapid triage and identification of cases that need urgent care thanks to the timely feedback that technologies such as digital patient monitoring enable [5,47,52,57,87,115,157]. Consequently, it can allow clinicians to prioritize by enabling them to focus more on patients that need assistance versus more stable patients [64,174]. Large scale rollout of such technologies also necessitates standardization, resulting in higher efficiency [177]. Unfortunately, there are still cases where such tools do not result in better efficiency in practice [94], mostly because of usability issues and difficulties with technology [108,110] and lack of EMR integration resulting in double work and reenter of data, which eliminates the efficacy gains achieved with the tools [44] and adds complexities to management [39]. Sometimes, efficiencies such as less in-clinic visits or phone calls come at the expense of a higher overall workload when there is no appropriate reimbursement [119]. Still, several studies suggested that the perceived higher efficiency can increase clinicians’ intention to use mHealth [61,78,107,132,140,150,181].

Improved collaboration and coordination among clinicians were among the identified facilitators [1,52,68,109,150,152,186]. Well-planned coordination of services can increase adoption, especially when several teams or sites are involved [2,8,88,162,176]. Better care coordination was also sometimes the result of the introduction of such technology [87,194], mainly when new multidisciplinary and integrated teams are formed [10,39,50,55,56,59,156,172,188], and peer support through second opinion and new models of shared decision making are created [40,57,93,102,137,153,159,189]. Conversely, some studies report that this can sometimes result in more pressure on clinicians, as the tools increase the possibilities to coordinate and communicate with other staff members, adding to their already high workload [46], and as interprofessional collaboration can be challenging [85], sometimes resulting in lack of trust or conflicting opinions [69,188]. The lack of coordination and collaboration was also seen as a barrier is some studies [71,84,141,182,185].

Poor technical skills and experience create uncertainties about how the technology may work, thus they can be major hurdles for adoption [37,55,59,67,81,127,143,153,179,184,191], whereas users’ familiarity with the technology may create confidence that may foster adoption [49,133,134,145,170]. The more IT-related knowledge and skills clinicians have, the lower their expected effort related to mHealth use becomes, resulting in an increase in their intention to use it [9,96,129,149]. Also, those with previous digital health experience are usually more willing to embrace mHealth than their counterparts that had not used such tools before [61,73,83,102,112,121,168]. However, it was also noted that in some cases, the fact that some clinicians use technology in their private life is not necessarily positively correlated with higher chances of technology adoption at the workplace [122,146]. It is suggested that the staff’s technical skills need to improve to enable the efficient use of such new technologies [72].

Smooth integration of mHealth may necessitate changes in staff’s roles and responsibilities [56,62,65,67,72], sometimes in the form of alignment of duties [48,86,152], role reassignment and redistribution [2,94], expanding existing staff members’ responsibilities [85], or even the creation of additional functions or staff numbers to cover some of the new tasks related to mHealth management [40,68,71,74,77,79,88,90,103]. It was noted that in some cases, the tools’ introduction resulted in a lack of clarity on roles demarcation [39,70,89,104,182], whereas clearly defined roles [61] and the presence of a local *champion* that can guide others on the technology use can contribute to a successful adoption [83,164]. The new tasks resulting from the use of such technologies are usually related to data analysis and interpretation [60,74,176], monitoring patient data and alerting the relevant staff accordingly [167], and also some other nonclinical tasks that were deemed sometimes undermining, such as equipment installation and troubleshooting [80,82,189]. The tools, moreover, allowed the delegation of more tasks to nursing staff in several studies, giving them more autonomy and empowering them in their role [78,169,183].

Leadership and institutional support were seen as a vital factor [5,9,95,127,132,134,157,182,188,192,196] and considered one of the most important facilitators of technology adoption [61,103,106,145,166,195]. Management support is crucial to facilitate the potential organizational changes that the new technologies entail, such as changes in roles and responsibilities [40], or changes in workflow [42,72,164], resource allocation [73,131,170], and proper training [111]. However, getting senior management support can be challenging at times [34,138,174], resulting in lack of recognition of clinicians’ activities taken with mHealth tools [59]; this may be explained by a lack of proper understanding of mHealth from the management side [178] or a false perception that such tools would detract staff from their real work [156]. Lack of organizational support can be a barrier that slows down adoption [71,86].

Organizational infrastructure is a basic prerequisite for mHealth success [87,91,116,126,179]. Factors such as access to the internet, equipment, and suitable space and power play a key role in whether or not clinicians would adopt such new technologies [4,47,51,69,77,93,124,127,153,154,163,169,181, 187]. Poor infrastructure may hinder adoption [35,70,82,83,151,172,193], as clinicians who have no access to suitable equipment may refrain from using mHealth [2].

Process standardization and planning may facilitate the tools’ uptake [45,73,86,88,149]. This can be achieved via streamlined procedures [39,80,160], process protocols and clear guidelines describing the practical details of integrating mHealth into clinical practice [40,168,185], and the presence of internal responsibility for facilitating this standardization [61,103,164]. Lack of planning or standardization of implementation strategies can hinder adoption as it can cause workflow challenges [5,44,46,67,82-84,155,167,189].

Staff nontechnical competence and qualifications also play a role in adoption [2,73,84,91,156,164,185]. Factors such as knowledge of medical terminology, a good command of the language in which the tool is offered, and the capacity to review large amounts of data and using the complex charts produced by some of these tools are paramount for successful adoption [4,36,109,176]. Given the shared decision-making models and higher collaboration enabled by mHealth, a potential hurdle may be the lack of confidence in the collaborators’ clinical competence [39,51]. Another subsequent difficulty is the fear of exposing knowledge gaps [105,108,137] or being marginalized and undermined [80]. Conversely, the fact that such tools enable less experienced clinicians to access clinical resources can also be a facilitator [69].

The ability to efficiently manage and interpret the large amounts of data generated by mHealth, such as interpreting complex charts, may also impact adoption [176]. Data management–related challenges can hinder the use of the tools [51,177]. Factors such as information overload [60,62,72] and the integration of the generated data in the existing workflow can be challenging [53,167,182]. Other data-related risks, such as adverse events reporting and further handling, may also hinder adoption [38]. Although such new technologies increase the potential to combine data to enhance patient monitoring and improve clinical decision making, some of the available tools do not give clinicians the flexibility to customize data reporting according to their specific needs [36,120,183]. Facilitators include availability and access to required data [66,77,100,150], higher efficiency of data analysis [74,108], better patient care management because of the timely availability of data [75,78,115,157], and the better ability to measure outcome [73].

mHealth requires a change of paradigm that mostly results in changes to clinical practice [48,59,65,92,186,189,192]. This is not necessarily a barrier to adoption; on the contrary, some studies show that clinicians are aware of the change that the technology entails and have already prepared themselves for it [139]. This paradigm shift is linked to factors such as patients’ self-monitoring and self-reporting, which necessitate new ways of treatment and care [10,72,162,185]. However, this redistribution of roles can sometimes be challenging [37,94,182]. For example, when a tool enables patients to access some of their test results before their care team, they can perceive this as an interference with established clinical practice [46].

Clinicians’ perceptions of mHealth’s impact on their autonomy and job security may also influence adoption [9,10,72,176,182,185,188]. Perceptions that the new tools compromise clinicians’ autonomy, for example, by making their patients’ treatment plans and outcomes more reachable to others and accordingly subject to more external control or criticism, may hinder adoption [62,116,130,140,141,186,188]. This can be a considerable barrier to adoption when care teams perceive the new technology as a threat to their own career and livelihood [55,72,141,151]. Equally, a tool has better chances of being adopted when perceived as a complement, not a substitute to clinicians’ role [62]. Some studies report that clinicians feel that they need to renegotiate their professional identities in the face of the *empowered and informed patient* that is sometimes seen as undermining the authority and credibility of the care teams [10,62,72,80,182]. Conversely, it was also reported that mHealth can empower clinicians and help them be more autonomous, positively impacting adoption [124].

Clinicians’ empowerment is tightly linked to the possibility of positively impacting their professional development and expertise because of the use of these new technologies, especially among nursing staff [62,72,124,162,169,188,189]. The educational benefits of mHealth for physicians can similarly encourage adoption [137,161]. The tools are perceived as enablers that prompt for best practice care, provide novel decision aids, and expand clinical knowledge [73,74,86,131].

Facilitating the adoption may be encouraged through proper incentives for clinicians [4,88,106,122,164]. Incentives such as awarding continuing medical education, adding mHealth use as an objective in employee appraisals, offering financial rewards through improved reimbursement schemes, and more clarity around medico-legal topics may encourage use [54,59,87,129,141,157,175,191].

Decision making can be a hurdle for adoption in the absence of a dedicated team or person responsible for digital health programs in the highly fragmented health care organizations [4,102,160,191]. This can also be an obstacle when the official decision makers do not involve practitioners in defining the aims and objectives of the introduction of an mHealth tool [43,71,72].

### Social and Organizational Factors: Patient Related

Improvements in the quality and efficiency of care may positively impact clinicians’ adoption of mHealth [3,5,44,52,61,63,64,66,69,83,86,100,107-109,120,122, 125,127,140,166,168,175,182,186,196]. Such tools can improve the quality of patient care through better information access, improved disease control, personalized treatment plans, and more proactive support [2,36,37,40,42,47,48,55-57,72,78,80, 88,92,93,103,114,115,119,130,157,159,160,162,174,184,188,190], although sometimes the perception that these new tools do not enhance patient care may hinder adoption [3,10]. Clinicians raised some concerns about the quality of patient reports, the possibility of overtreatment, or false positives being reported through the tools [38,183].

The impact of mHealth use on patient-clinician communication can also influence clinicians’ adoption decision [47,88, 128,163,185,195]. This factor can be considered a facilitator when the tool enhances communication [3,5,50,58, 59,66,67,75,77,78,82,115,150,172,174,175,182,189], but it can also be considered a barrier when clinicians perceive the tool as an obstruction to their communications with their patients [10,43,46,55,89,180]. Clinicians’ concerns about digital communications are mostly about the loss of human contact, breaching patient privacy, medico-legal issues, unprofessional image, and patient’s overreliance [41,72,79,80,90,156,160,190]. It was also emphasized that such tools should complement rather than replace face-to-face treatment and therapy [81,86,91].

Improving patients’ access to care by removing time and space constraints may encourage clinicians’ adoption [1,3, 5,8,43,64,73,82,129,130,156,162,174,182,184,190]. This is especially true when the tools allow underserved patients, those residing in rural or remote areas, or suffering from a chronic condition, to access health care services [4,34, 39,47,54,55,67,75,81,93,102,123,159,178,194]. mHealth may enable better access by eliminating or reducing travel burden [2,4,60,62,78,91,169,187,195].

From the clinicians’ perspective, patient consent, comfort, and preference play an important role in adoption [2,9,39,49,90,104,122,123,132,153,185,192]. Several elements could impact patients’ preferences, such as age, the complexity of the condition, access to technology, tech-savviness, or privacy concerns [1,43,52,53,60,174,176,180,184,191,195]. In some cases, patients may feel more comfortable to use mHealth than face-to-face care when they are treated for sensitive conditions such as HIV or sexual health [3,163].

It was reported that clinicians believe that mHealth may not be applicable or appropriate to all sorts of patients [9, 61,68,88,90,91,103,189]. Consequently, it is important to have balanced selection criteria [71,79,89,162,182]. Some perceive the tools to be more appropriate for chronic and unstable patients that need more attention to support their stabilization [41,66], although others deemed the technology inappropriate for those with physical or psychological impairments, severely ill patients, and the ones unable to properly use technology [62,73,75,81,86,104,185]. However, it is important to note that restrictive inclusion criteria might prevent some patients who need the service from accessing it [71].

Clinicians are more likely to adopt mHealth when it empowers and engages patients, giving them more autonomy and assurance about their disease or condition management [5,34, 44,48,62,73,77,78,88,89,115,157,166,175,180]. However, more evidence is needed to confirm such a positive impact on patient empowerment [128]. It is also worth noting that in some contexts, patients may initially be anxious from such increased empowerment, as they are afraid of taking responsibility. However, research suggests that their confidence may develop with long-term support [41]. Patient engagement might also be a barrier if they perceive mHealth as a burden, or when they do not feel that they need it [70,71]

Safety concerns can impact intention to use [72,103]. Perceived risks to patient safety can be a barrier to adoption, mainly when clinicians are concerned about factors such as device contamination, system reliability, clinical content accuracy, and self-diagnosing [10,63,81,179,190]. Conversely, adoption may be encouraged when mHealth increases patient safety through timeliness, early detection, or clear documentation [44,66,78,86,109,140,176,188,195].

The digital divide, defined in the Oxford dictionary [198] as “the gulf between those who have ready access to computers and the Internet, and those who do not,” also plays a role in the adoption. Clinicians are concerned about patients that might be marginalized because they lack access to technology, the elderly that do not use smartphones, those who have literacy issues, or a lower living standard [49,53,55,60,73,75,79,81,84,120, 123,138,185, 191]. A study reported that nurses do not see patients’ age as a barrier [62].

Better patient education and awareness because of the use of mHealth tools may encourage adoption [52,53,60,75, 86,88,162,164,168,183,190,199]. However, clinicians were concerned that the convenience of the new tools might result in patients’ overreliance on their practitioner support [3,41,78,182]. Service abuse might occur when they overutilize the tool or try to access their care team after hours [156,169], or if they become too dependent on technology and fail to seek medical help in case of emergency [62,77].

Clinicians also pinpointed that data and surveillance-related anxiety might hinder adoption [140,190]. Patients might worry excessively because of the large amounts of data available through mHealth [46,128] or might feel watched because of the constant monitoring enabled by such tools [62,78]. Furthermore, the sustainability of mHealth services depends on several factors, such as the willingness of patients to keep using it [5], which might depend on the long-term availability of funding [167] and clinicians’ long-term commitment [169]. It is also notable that clinicians might play a gatekeeping role for mHealth directly impacting adoption [97]; this may be driven by their willingness to protect their patients from any added burden [180].

### Other Social and Organizational Factors

Concerns related to data privacy and security can be a barrier to adoption [9,39,67,84,95,108,117,132,143,158,174,177,185, 191,192]. Worries about confidentiality, fear of inappropriate data use, anonymity, and medico-legal risks were the main drivers of this [2,3,10,37,47,49,52,53,59,98,100,102,110, 123,128,130,152,160,163,167,178,182-184,189,193]. mHealth was perceived to be more prone to data security issues compared with other forms of digital health tools because of its portability and accessibility from personal devices [73,77,127]. Therefore, it is deemed important to have a safe tool that protects the data provided in it [38,61], work out liability issues in advance [60,131], provide data privacy training to clinicians [138], and have clearer legal guidance [161,162]. Interestingly, some studies reported that privacy concerns are not a key barrier to adoption, given that some clinicians that have high privacy concerns might still have high usage intentions [125,142,149].

Policy and regulations mostly related to malpractice protection, licensing, and credentialing, in addition to costs and reimbursement issues, can certainly impact clinicians’ adoption of mHealth tools [9,10,37,61,73,95,121,123,130,141,151]. Unsuitable or inconsistent regulations and ambiguous policies [5,35,63,155,159,179], restrictive directives [64,102], lack of policies and protocols [34,47,51,67,104,161,162], and lack of governmental coordination [4] may hinder adoption. Clear and appropriate regulations and guidelines [92,122,124, 146,168,178,190,193] and more proactive involvement of governments and medical professional associations [143,145,157] are essential for efficient adoption and sustainable services.

Clinicians’ perceptions and attitudes toward technology may impact their decision to adopt the tools [5,35,69,107, 118,127,134,136,138,173]. Those who are lacking familiarity with mobile technologies, are resistant to change, or are risk averse may not use them [34,60,67, 85,108,117,174,186,195]; also, there is the perception that using a mobile phone at work may seem unprofessional [63,124,190]. Those with positive attitudes toward technology are more likely to adopt new tools [97,113]. In addition, their perception and attitudes toward mHealth specifically also affect their intention to use it [10,95,109,111,130,132,133,147,178,185,188]. Clinicians with negative attitudes toward mHealth are more reluctant to use it [143,155]. This is sometimes because of their perception that it will add to their workload [37,83], their uncertainty to what the introduction of such new tools would mean for their workflow [55,104,153,166], or their perception that it invades their privacy [47,65,77,161,188,190]. It is also important to note that personal attributes such as adaptability and the readiness to try new things may similarly impact the adoption decision [59,100,157].

The organizational culture and context can also impact the clinician’s intention to use the tools [95,151,168], although some studies reported that it is not a meaningful barrier [130]. Prohibitive or unclear expectations around mobile phone use in the workplace may discourage adoption [42,63,76, 124,161,165,170,179]. Furthermore, an organizational culture that is resistant to change or risk averse may hinder the implementation of such new technologies [45,86,148,176]. A cultural shift might be needed to enable and foster the acceptance of mHealth use at the workplace and transition from paper-based systems to more use of digital tools [37,145,193].

Peer influence and endorsement are other factors that might impact clinicians’ trust in mHealth and, accordingly, their adoption decision [2,9,125,142,170,175,182,196]. Equally, those who are *change-resistant* may also impact early adopters negatively [181]. Recommendations by reliable bodies such as scientific societies, renowned health care organizations such as the National Health Service, opinion leaders, internal champions, direct managers, or senior colleagues that promote the tools may foster adoption [38,51,61,67,99,105,128,146,161,163].

Financial aspects are typically barriers to adoption [70,101,152,168]. Lack of proper funding [36,71,86, 143,151,156,160,164,167] and compensation or reimbursement problems [2,3,5,9,35,51,54,56,60,61,84,100,119,123,128-130, 184,186] usually hinder clinicians’ intention to use mHealth. In addition, low awareness of existing reimbursement schemes [102] and overcomplicated or inconsistent payment systems [39,64,67,141,159,162] may also be a barrier. Conversely, a suitable payment model and health insurance coverage may encourage adoption [8,67,68,77,92,122,131,182,195]. It is noteworthy that 1 study found that the financial disadvantages because of funding and reimbursement issues were compensated by the lower travel costs and higher efficiency generated by mHealth use [91].

Reducing organizational costs may positively impact clinicians’ adoption as it helps them achieve budget efficiency [1,2,5,91,132,147,159,182]. However, uncertainties around cost-effectiveness [34,41,73,78,114] and the actual tool’s or service’s charge and affordability [9,38,39,51,53,55, 60,61,63,64,67,84,110,123,142,147,156,191] may hinder adoption.

Clinicians’ perception that the timeliness and amount of the data generated by mHealth can enhance the evidence for benefit is a facilitator [85,89,108,109,115,159,169]. However, the perceived lack of a solid evidence base and proof of concept for clinical benefit resulting from mHealth use is considered a barrier to adoption [5,43,60,69,73,84,160,162,175,188]. There is a need for more research about the outcomes of such technologies use in clinical practice to help foster adoption [55,155,186].

The lack of awareness of mHealth tools may hinder adoption [73,120,151,175]. Active promotion of the tools’ existence and objectives [9,55,56,150,156], and their benefits and impact on patient outcome may encourage their use [2,38,43,61,89,131,149,168,180,182].

Engaging users in the development, planning, and implementation phases may positively impact their adoption decision [68,86,135,169,172,183]. Enabling user feedback [38,52], collaborative involvement, and codesign [10,61] have shown to encourage adoption. Unfortunately, in some cases, clinicians are hardly asked for their input or involvement even though mHealth is one of their work tools [72,182].

### Moderating Factors

Some of the included studies reported that moderating factors, such as age, gender, specialty, and years of professional experience, may have an impact on clinicians’ adoption intentions [38,106]. However, other studies concluded that such moderating factors do not necessarily influence mHealth usage [117,127,196].

Younger clinicians typically have a more positive attitude toward such new technologies compared with their older counterparts [59,73,76,81,100,118,122,125,135,138], although some studies established that the age-gap does not play a role in adoption [142,143]. Also, clinicians with previous digital health experience seem to have more favorable attitudes toward mHealth adoption compared with their counterparts that have no previous experience [120,122,142].

Gender does not seem to be consistently reported as a moderating factor; 2 studies reported that female clinicians are more likely to accept the tools than their male counterparts [134,143], whereas 1 study reported the reverse [133]. The years of professional experience seem to negatively impact adoption [111,118].

### Conclusions and Implications

**Figure 5:**
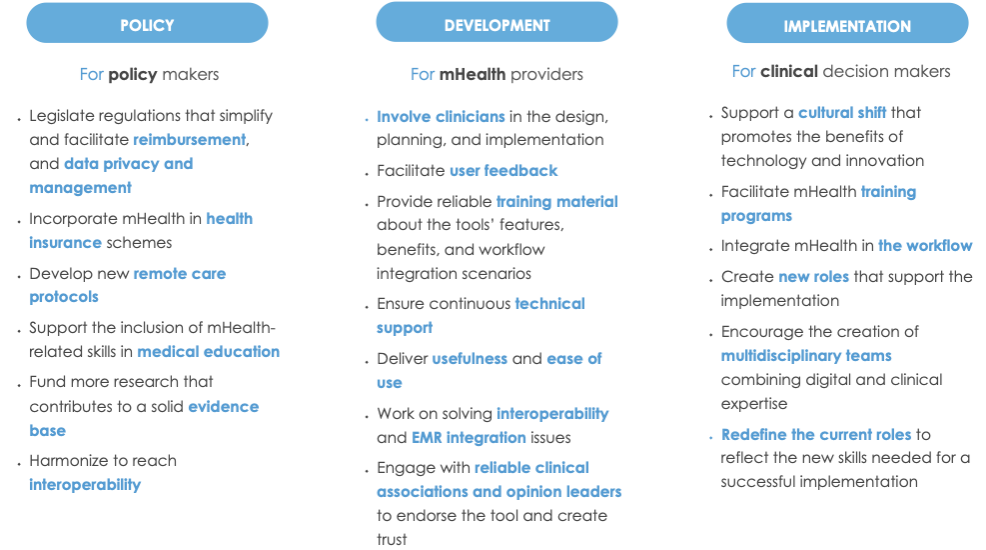
Implications for social and organizational practices. EMR: electronic medical record.

The systematic review findings indicate important guidelines and areas that must be targeted regarding social and organizational practices to promote and foster clinicians’ adoption of mHealth tools successfully. As shown in Figure 5, these implications can be split into 3 categories to address the actions needed from 3 key stakeholders: policymakers, mHealth providers, and clinical decision makers.

Policymakers can play a crucial role in unleashing the potential of mHealth in clinical practice. This can be achieved through regulations that simplify and facilitate reimbursement and address data privacy and management issues. Incorporating mHealth in health insurance schemes can help solve the cost and payment barriers and encourage not only clinicians’ adoption but also patients’ use. Developing new remote care protocols may help practitioners standardize these new services and better integrate them into their clinical practice. It is also important to support the inclusion of mHealth-related skills in official medical education to ensure that new graduates will be equipped with all the necessary capabilities to successfully run such new technologies. Funding more research that contributes to a solid evidence base about the clinical and efficiency benefits and the added value of the new tools can foster their acceptance. Coordinating the different stakeholders to streamline and harmonize technology in a way that helps reach interoperability would also ensure the successful implementation of such tools and solve the issue of additional workload that sometimes results from a double entry in the different systems.

Such policy implications echo the findings of other researchers. For example, Davis et al [183] addressed the barriers related to unclear legal liability and system interoperability. Gagnon et al [9] also discussed the importance of cost-related barriers and the need to address them.

Providers and developers of mHealth tools should always proactively involve clinicians in the design, planning, and implementation of their services to ensure that it fits well into clinical practice. Facilitating user feedback is key to warrant the relevance and sustainability of the tool. Providing reliable training material about the tools’ features, benefits, and workflow integration scenarios may help clinicians better integrate the new tools into their daily practice. Delivering tools that are useful and ease of use, and ensuring continuous technical support, is crucial for smooth day-to-day usage and to overcome any technical issues that might push users to abandon the tool. Working on solving interoperability and EMR integration issues would help them emphasize the efficiency gains resulting from their services that are sometimes compromised because of the burden of double data entry when systems are not integrated properly. Furthermore, engaging with reliable clinical associations and opinion leaders to endorse the tool can help create trust and accelerate the adoption.

Recommendations for providers and developers of mHealth tools are aligned with Brewster et al [10], Davis et al [183], and Radhakrishnan et al [182] conclusions on the importance of the inclusion of clinicians in the development process to improve acceptance. Davis et al [183] also shed light on the importance of system interoperability and EHR integration to facilitate adoption.

Clinical decision makers in hospitals and clinics need to support a cultural shift that promotes the benefits of technology and innovation to encourage their staff to change their traditional ways of working and embrace the new modalities. Facilitating mHealth training programs may help clinical staff acquire the new skills needed to successfully adopt the new tools. In addition, integrating mHealth in the clinical workflow is key to avoid that the tools become more of a hurdle to the staff. Encouraging the creation of multidisciplinary teams combining digital and clinical expertise and redefining the current roles to reflect the new skills needed to run the new technologies may contribute to a successful implementation. In some cases, the creation of new roles that support the implementation might be necessary.

In terms of implications for clinical decision makers, our findings are congruent with those of Duennebeil et al [149], who emphasized the importance of establishing standards and treatment processes and training programs that would enable the adoption of such new tools. Training, promotion, and redefinition of roles were also highlighted by Brewster et al [10] and Radhakrishnan et al [182].

### Limitations and Recommendations for Future Research

Although this study contributes to the understanding of the factors impacting clinicians’ adoption of mHealth, some limitations must be acknowledged. This review may not have included relevant studies that were not indexed in the searched databases, written in a language other than English, and grey literature searches that could have also allowed the identification of additional relevant insights. However, this study meant to concentrate on peer-reviewed scientific papers.

Moreover, this analysis only considered published studies, and no further contacts were made with the papers’ authors to obtain extra information or to validate our thematic analysis. Consequently, it is possible that other mHealth adoption factors might have been missed. Future reviews could include studies in other languages to have a better grasp of any interregional or intercultural differences, and to have more studies in developed countries.

## Appendix

Multimedia Appendix 1 Screenshot of Rayyan QCRI (Qatar Computing Research Institute).

Multimedia Appendix 2 Critical Appraisal Skills Program checklist.

Multimedia Appendix 3 Critical appraisal of the included studies.

Multimedia Appendix 4 Phases of thematic analysis after Braun and Clarke.

## Abbreviations

eHealth: electronic health
EHR: electronic health record
EMR: electronic medical record
IT: information technology
mHealth: mobile health
PICO: participants, intervention, comparators, and outcome framework
PRISMA: Preferred Reporting Items for Systematic Review and Meta-Analysis
TAM: Technology Acceptance Model
UTAUT: unified theory of acceptance and use of technology

## References

[1] Armstrong AW, Kwong MW, Chase EP, Ledo L, Nesbitt TS, Shewry SL (2012). Teledermatology operational considerations, challenges, and benefits: the referring providers' perspective. Telemed J E Health.

[2] Avey JP, Hobbs RL (2013). Dial in: fostering the use of telebehavioral health services in frontier Alaska. Psychol Serv.

[3] Anderson K, Francis T, Ibanez-Carrasco F, Globerman J (2017). Physician's perceptions of telemedicine in HIV care provision: a cross-sectional web-based survey. JMIR Public Health Surveill.

[4] Bhatta R, Aryal K, Ellingsen G (2015). Opportunities and challenges of a rural-telemedicine program in Nepal. J Nepal Health Res Counc.

[5] Mileski M, Kruse CS, Catalani J, Haderer T (2017). Adopting telemedicine for the self-management of hypertension: systematic review. JMIR Med Inform.

[6] Pavel M, Jimison HB, Wactlar HD, Hayes TL, Barkis W, Skapik J, Kaye J (2013). The role of technology and engineering models in transforming healthcare. IEEE Rev Biomed Eng.

[7] Payne KF, Wharrad H, Watts K (2012). Smartphone and medical related App use among medical students and junior doctors in the United Kingdom (UK): a regional survey. BMC Med Inform Decis Mak.

[8] Choi WS, Park J, Choi JY, Yang J (2019). Stakeholders' resistance to telemedicine with focus on physicians: utilizing the Delphi technique. J Telemed Telecare.

[9] Gagnon MP, Ngangue P, Payne-Gagnon J, Desmartis M (2016). m-Health adoption by healthcare professionals: a systematic review. J Am Med Inform Assoc.

[10] Brewster L, Mountain G, Wessels B, Kelly C, Hawley M (2014). Factors affecting front line staff acceptance of telehealth technologies: a mixed-method systematic review. J Adv Nurs.

[11] Hussein R, Khalifa A (2012). Telemedicine in Egypt: SWOT analysis and future trends. GMS Medizinische Inform Biometrie und Epidemiol.

[12] Moffatt JJ, Eley DS (2011). Barriers to the up-take of telemedicine in Australia--a view from providers. Rural Remote Health.

[13] Walter Z, Lopez MS (2008). Physician acceptance of information technologies: role of perceived threat to professional autonomy. Decis Support Syst.

[14] Xue Y, Liang H, Mbarika V, Hauser R, Schwager P, Getahun MK (2015). Investigating the resistance to telemedicine in Ethiopia. Int J Med Inform.

[15] Gagnon MP, Desmartis M, Labrecque M, Car J, Pagliari C, Pluye P, Frémont P, Gagnon J, Tremblay N, Légaré F (2012). Systematic review of factors influencing the adoption of information and communication technologies by healthcare professionals. J Med Syst.

[16] World Health Organization (2011). mHealth: New Horizons for Health through Mobile Technologies.

[17] Rogers EM (2003). Diffusion of Innovations. Fifth Edition.

[18] Straub ET (2009). Understanding technology adoption: theory and future directions for informal learning. Rev Educ Res.

[19] Goffman E (1956). The Presentation of Self in Everyday Life.

[20] Pinch T (2010). The invisible technologies of Goffman's sociology from the merry-go-round to the internet. Technol Cult.

[21] Garfinkel H (1967). Studies in Ethnomethodology.

[22] Leonardi PM, Raza M, Jain S (2017). Methodological guidelines for the study of materiality and affordances. Routledge Companion to Qualitative Research in Organization Studies.

[23] Bélanger F, Watson-Manheim MB (2006). Virtual teams and multiple media: Structuring media use to attain strategic goals. Group Decis Negot.

[24] Chudoba K, Wynn E, Lu M, Watson-Manheim MB (2005). How virtual are we? Measuring virtuality and understanding its impact in a global organization. Inf Syst J.

[25] Jacob C, Sanchez-Vazquez A, Ivory C (2019). Clinicians' role in the adoption of an oncology decision support app in Europe and its implications for organizational practices: qualitative case study. JMIR Mhealth Uhealth.

[26] Moher D, Liberati A, Tetzlaff J, Altman DG, PRISMA Group (2009). Preferred reporting items for systematic reviews and meta-analyses: the PRISMA statement. PLoS Med.

[27] Higgins JP, Green S (2011). Cochrane Handbook for Systematic Reviews of Interventions.

[28] Jacob C, Ivory C, Sánchez-Vázquez A (2018). CRD Database.

[29] Schardt Connie, Adams Martha B, Owens Thomas, Keitz Sheri, Fontelo Paul (2007). Utilization of the PICO framework to improve searching PubMed for clinical questions. BMC Med Inform Decis Mak.

[30] Merriam-Webster (1997). Merriam Webster's Collegiate Dictionary.

[31] Ouzzani M, Hammady H, Fedorowicz Z, Elmagarmid A (2016). Rayyan-a web and mobile app for systematic reviews. Syst Rev.

[32] (2018). Critical Appraisal Skills Program.

[33] Braun V, Clarke V (2006). Using thematic analysis in psychology. Qual Res Psychol.

[34] Catan G, Espanha R, Mendes RV, Toren O, Chinitz D (2015). Health information technology implementation - impacts and policy considerations: a comparison between Israel and Portugal. Isr J Health Policy Res.

[35] Chiang KF, Wang HH, Chien IK, Liou JK, Hung CL, Huang CM, Yang FY (2015). Healthcare providers' perceptions of barriers in implementing of home telecare in Taiwan: a qualitative study. Int J Med Inform.

[36] Chung C, Cook J, Bales E, Zia J, Munson SA (2015). More than telemonitoring: health provider use and nonuse of life-log data in irritable bowel syndrome and weight management. J Med Internet Res.

[37] de Souza CH, Morbeck RA, Steinman M, Hors CP, Bracco MM, Kozasa EH, Leão ER (2017). Barriers and benefits in telemedicine arising between a high-technology hospital service provider and remote public healthcare units: a qualitative study in Brazil. Telemed J E Health.

[38] de Vries ST, Wong L, Sutcliffe A, Houÿez F, Ruiz CL, Mol PG, IMI Web-RADR Work Package 3b Consortium (2017). Factors influencing the use of a mobile app for reporting adverse drug reactions and receiving safety information: a qualitative study. Drug Saf.

[39] Egerton T, Nelligan R, Setchell J, Atkins L, Bennell KL (2017). General practitioners' perspectives on a proposed new model of service delivery for primary care management of knee osteoarthritis: a qualitative study. BMC Fam Pract.

[40] Esterle L, Mathieu-Fritz A (2013). Teleconsultation in geriatrics: impact on professional practice. Int J Med Inform.

[41] Fairbrother P, Ure J, Hanley J, McCloughan L, Denvir M, Sheikh A, McKinstry B, Telescot programme team (2014). Telemonitoring for chronic heart failure: the views of patients and healthcare professionals - a qualitative study. J Clin Nurs.

[42] Farrell M (2016). Use of iPhones by nurses in an acute care setting to improve communication and decision-making processes: qualitative analysis of nurses' perspectives on iPhone use. JMIR Mhealth Uhealth.

[43] Flynn D, Gregory P, Makki H, Gabbay M (2009). Expectations and experiences of eHealth in primary care: a qualitative practice-based investigation. Int J Med Inform.

[44] Giraldo L, Schachner B, Luna D, Benítez S (2018). Exploring nurses' perceptions and expectations toward a BCMA implementation using a mobile app and workstations as a change management strategy. Stud Health Technol Inform.

[45] Goedken CC, Moeckli J, Cram PM, Reisinger HS (2017). Introduction of Tele-ICU in rural hospitals: changing organisational culture to harness benefits. Intensive Crit Care Nurs.

[46] Grünloh C, Cajander A, Myreteg G (2016). 'The Record is Our Work Tool!'- physicians' framing of a patient portal in Sweden. J Med Internet Res.

[47] Han KJ, Subramanian R, Cameron GT (2019). Listen before you leap: Sri Lankan health professionals' perspectives on m-health. Health Informatics J.

[48] Hanley J, Ure J, Pagliari C, Sheikh A, McKinstry B (2013). Experiences of patients and professionals participating in the HITS home blood pressure telemonitoring trial: a qualitative study. BMJ Open.

[49] Hanna L, May C, Fairhurst K (2012). The place of information and communication technology-mediated consultations in primary care: GPs' perspectives. Fam Pract.

[50] Hines M, Lincoln M, Ramsden R, Martinovich J, Fairweather C (2015). Speech pathologists' perspectives on transitioning to telepractice: what factors promote acceptance?. J Telemed Telecare.

[51] James S, Perry L, Gallagher R, Lowe J (2016). Diabetes educators: perceived experiences, supports and barriers to use of common diabetes-related technologies. J Diabetes Sci Technol.

[52] Jarvis-Selinger S, Bates J, Araki Y, Lear SA (2011). Internet-based support for cardiovascular disease management. Int J Telemed Appl.

[53] Jimbo M, Shultz CG, Nease DE, Fetters MD, Power D, Ruffin MT (2013). Perceived barriers and facilitators of using a Web-based interactive decision aid for colorectal cancer screening in community practice settings: findings from focus groups with primary care clinicians and medical office staff. J Med Internet Res.

[54] Armstrong AW, Kwong MW, Ledo L, Nesbitt TS, Shewry SL (2011). Practice models and challenges in teledermatology: a study of collective experiences from teledermatologists. PLoS One.

[55] Kayyali R, Hesso I, Mahdi A, Hamzat O, Adu A, Nabhani Gebara S (2017). Telehealth: misconceptions and experiences of healthcare professionals in England. Int J Pharm Pract.

[56] Khan NU, Rasheed S, Sharmin T, Ahmed T, Mahmood SS, Khatun F, Hanifi S, Hoque S, Iqbal M, Bhuiya A (2015). Experience of using mHealth to link village doctors with physicians: lessons from Chakaria, Bangladesh. BMC Med Inform Decis Mak.

[57] Kim JW, Tiyyagura G, Langhan M (2019). A qualitative analysis of general emergency medicine providers' perceptions on pediatric emergency telemedicine. Pediatr Emerg Care.

[58] Kopanitsa G, Yampolsky V (2016). Exploring barriers and opportunities for adoption of web portals in Russia. Stud Health Technol Inform.

[59] Tintorer DL, Domínguez JM, Pujol-Rivera E, Beneyto SF, Tuduri XM, Saigí-Rubió F (2018). Keys to success of a community of clinical practice in primary care: a qualitative evaluation of the ECOPIH project. BMC Fam Pract.

[60] Levine M, Richardson JE, Granieri E, Reid MC (2014). Novel telemedicine technologies in geriatric chronic non-cancer pain: primary care providers' perspectives. Pain Med.

[61] Lord S, Moore SK, Ramsey A, Dinauer S, Johnson K (2016). Implementation of a substance use recovery support mobile phone app in community settings: qualitative study of clinician and staff perspectives of facilitators and barriers. JMIR Ment Health.

[62] MacNeill V, Sanders C, Fitzpatrick R, Hendy J, Barlow J, Knapp M, Rogers A, Bardsley M, Newman SP (2014). Experiences of front-line health professionals in the delivery of telehealth: a qualitative study. Br J Gen Pract.

[63] McNally G, Frey R, Crossan M (2017). Nurse manager and student nurse perceptions of the use of personal smartphones or tablets and the adjunct applications, as an educational tool in clinical settings. Nurse Educ Pract.

[64] Merchant KA, Ward MM, Mueller KJ, Rural Health Research & Policy Centers, Rural Policy Research Institute‚ RUPRI Center for Rural Health Policy Analysis‚ University of Iowa College of Public Health‚ Department of Health Management and Policy (2015). Hospital views of factors affecting telemedicine use. Rural Policy Brief.

[65] Bagot KL, Cadilhac DA, Bladin CF, Watkins CL, Vu M, Donnan GA, Dewey HM, Emsley HC, Davies DP, Day E, Ford GA, Price CI, May CR, McLoughlin AS, Gibson JM, Lightbody CE, VSTASTUTE investigators (2017). Integrating acute stroke telemedicine consultations into specialists' usual practice: a qualitative analysis comparing the experience of Australia and the United Kingdom. BMC Health Serv Res.

[66] Moharra M, Almazán C, Decool M, Nilsson A, Allegretti N, Seven M (2015). Implementation of a cross-border health service: physician and pharmacists' opinions from the epSOS project. Fam Pract.

[67] Molfenter T, Boyle M, Holloway D, Zwick J (2015). Trends in telemedicine use in addiction treatment. Addict Sci Clin Pract.

[68] Molleda L, Bahamon M, St George SM, Perrino T, Estrada Y, Herrera DC, Pantin H, Prado G (2017). Clinic personnel, facilitator, and parent perspectives of eHealth Familias Unidas in primary care. J Pediatr Health Care.

[69] Moloczij N, Mosley I, Moss KM, Bagot KL, Bladin CF, Cadilhac DA (2015). Is telemedicine helping or hindering the delivery of stroke thrombolysis in rural areas? A qualitative analysis. Intern Med J.

[70] Morrow S, Daines L, Wiener-Ogilvie S, Steed L, McKee L, Caress A, Taylor SJ, Pinnock H (2017). Exploring the perspectives of clinical professionals and support staff on implementing supported self-management for asthma in UK general practice: an IMPART qualitative study. NPJ Prim Care Respir Med.

[71] Odeh B, Kayyali R, Nabhani-Gebara S, Philip N (2014). Implementing a telehealth service: nurses' perceptions and experiences. Br J Nurs.

[72] Öberg U, Orre CJ, Isaksson U, Schimmer R, Larsson H, Hörnsten Å (2018). Swedish primary healthcare nurses' perceptions of using digital eHealth services in support of patient self-management. Scand J Caring Sci.

[73] Puszka S, Dingwall KM, Sweet M, Nagel T (2016). E-Mental health innovations for aboriginal and Torres Strait Islander Australians: a qualitative study of implementation needs in health services. JMIR Ment Health.

[74] Rothstein JD, Jennings L, Moorthy A, Yang F, Gee L, Romano K, Hutchful D, Labrique AB, LeFevre AE (2016). Qualitative assessment of the feasibility, usability, and acceptability of a mobile client data app for community-based maternal, neonatal, and child care in rural Ghana. Int J Telemed Appl.

[75] Sandberg J, Trief PM, Izquierdo R, Goland R, Morin PC, Palmas W, Larson CD, Strait JG, Shea S, Weinstock RS (2009). A qualitative study of the experiences and satisfaction of direct telemedicine providers in diabetes case management. Telemed J E Health.

[76] Beauregard P, Arnaert A, Ponzoni N (2017). Nursing students' perceptions of using smartphones in the community practicum: a qualitative study. Nurse Educ Today.

[77] Schneider T, Panzera AD, Martinasek M, McDermott R, Couluris M, Lindenberger J, Bryant C (2016). Physicians' perceptions of mobile technology for enhancing asthma care for youth. J Child Health Care.

[78] Seto E, Leonard KJ, Cafazzo JA, Barnsley J, Masino C, Ross HJ (2012). Perceptions and experiences of heart failure patients and clinicians on the use of mobile phone-based telemonitoring. J Med Internet Res.

[79] Sharma U, Barnett J, Clarke M (2010). Clinical users' perspective on telemonitoring of patients with long term conditions: understood through concepts of Giddens's structuration theory & consequence of modernity. Stud Health Technol Inform.

[80] Sharma U, Clarke M (2014). Nurses' and community support workers' experience of telehealth: a longitudinal case study. BMC Health Serv Res.

[81] Sinclair C, Holloway K, Riley G, Auret K (2013). Online mental health resources in rural Australia: clinician perceptions of acceptability. J Med Internet Res.

[82] Sturesson L, Groth K (2018). Effects of the digital transformation: qualitative study on the disturbances and limitations of using video visits in outpatient care. J Med Internet Res.

[83] Taylor J, Coates L (2015). Caring from a distance: the role of telehealth. Nurs Times.

[84] van Gaalen JL, van Bodegom-Vos L, Bakker MJ, Snoeck-Stroband JB, Sont JK (2016). Internet-based self-management support for adults with asthma: a qualitative study among patients, general practitioners and practice nurses on barriers to implementation. BMJ Open.

[85] Varsi C, Ekstedt M, Gammon D, Børøsund E, Ruland CM (2015). Middle managers' experiences and role in implementing an interactive tailored patient assessment eHealth intervention in clinical practice. Comput Inform Nurs.

[86] Varsi C, Ekstedt M, Gammon D, Ruland CM (2015). Using the consolidated framework for implementation research to identify barriers and facilitators for the implementation of an internet-based patient-provider communication service in five settings: a qualitative study. J Med Internet Res.

[87] Bello AK, Molzahn AE, Girard LP, Osman MA, Okpechi IG, Glassford J, Thompson S, Keely E, Liddy C, Manns B, Jinda K, Klarenbach S, Hemmelgarn B, Tonelli M (2017). Patient and provider perspectives on the design and implementation of an electronic consultation system for kidney care delivery in Canada: a focus group study. BMJ Open.

[88] Vest BM, Hall VM, Kahn LS, Heider AR, Maloney N, Singh R (2017). Nurse perspectives on the implementation of routine telemonitoring for high-risk diabetes patients in a primary care setting. Prim Health Care Res Dev.

[89] Wilhelmsen M, Høifødt RS, Kolstrup N, Waterloo K, Eisemann M, Chenhall R, Risør MB (2014). Norwegian general practitioners' perspectives on implementation of a guided web-based cognitive behavioral therapy for depression: a qualitative study. J Med Internet Res.

[90] Wynn R, Bergvik S, Pettersen G, Fossum S (2012). Clinicians' experiences with videoconferencing in psychiatry. Stud Health Technol Inform.

[91] Zilliacus E, Meiser B, Lobb E, Dudding TE, Barlow-Stewart K, Tucker K (2010). The virtual consultation: practitioners' experiences of genetic counseling by videoconferencing in Australia. Telemed J E Health.

[92] Carlisle K, Warren R (2013). A qualitative case study of telehealth for in-home monitoring to support the management of type 2 diabetes. J Telemed Telecare.

[93] Cary MP, Spencer M, Carroll A, Hand DH, Amis K, Karan E, Cannon RF, Morgan MS, Hoenig HM (2016). Benefits and challenges of delivering tele-rehabilitation services to rural veterans. Home Healthc Now.

[94] Casey M, Shaw S, Swinglehurst D (2017). Experiences with online consultation systems in primary care: case study of one early adopter site. Br J Gen Pract.

[95] Ghani MK, Jaber MM (2015). Willingness to adopt telemedicine in major iraqi hospitals: a pilot study. Int J Telemed Appl.

[96] Duplaga M (2016). Searching for a role of nursing personnel in developing landscape of eHealth: factors determining attitudes toward key patient empowering applications. PLoS One.

[97] Gagnon MP, Orruño E, Asua J, Abdeljelil AB, Emparanza J (2012). Using a modified technology acceptance model to evaluate healthcare professionals' adoption of a new telemonitoring system. Telemed J E Health.

[98] Hackl WO, Hoerbst A, Duftschmid G, Gall W, Janzek-Hawlat S, Jung M, Woertz K, Dorda W, Ammenwerth E (2014). Crucial factors for the acceptance of a computerized national medication list: insights into findings from the evaluation of the Austrian e-Medikation pilot. Appl Clin Inform.

[99] Hao H, Padman R (2018). An empirical study of opinion leader effects on mobile technology implementation by physicians in an American community health system. Health Informatics J.

[100] Holderried M, Hoeper A, Holderried F, Blumenstock G, Ernst C, Tropitzsch A (2018). Attitudes toward e-Health: the otolaryngologists' point of view. Telemed J E Health.

[101] Jefee-Bahloul H, Duchen D, Barkil-Oteo A (2016). Attitudes towards implementation of Store-and-Forward telemental health in humanitarian settings: survey of Syrian healthcare providers. Telemed J E Health.

[102] Jetty A, Moore MA, Coffman M, Petterson S, Bazemore A (2018). Rural family physicians are twice as likely to use telehealth as urban family physicians. Telemed J E Health.

[103] Jury SC, Walker AM, Kornberg AJ (2013). The introduction of web-based video-consultation in a paediatric acute care setting. J Telemed Telecare.

[104] Kato NP, Johansson P, Okada I, de Vries AE, Kinugawa K, Strömberg A, Jaarsma T (2015). Heart failure telemonitoring in Japan and Sweden: a cross-sectional survey. J Med Internet Res.

[105] Kifle M, Payton FC, Mbarika V, Meso P (2010). Transfer and adoption of advanced information technology solutions in resource-poor environments: the case of telemedicine systems adoption in Ethiopia. Telemed J E Health.

[106] Adenuga KI, Iahad NA, Miskon S (2017). Towards reinforcing telemedicine adoption amongst clinicians in Nigeria. Int J Med Inform.

[107] Kim S, Lee KH, Hwang H, Yoo S (2016). Analysis of the factors influencing healthcare professionals' adoption of mobile electronic medical record (EMR) using the unified theory of acceptance and use of technology (UTAUT) in a tertiary hospital. BMC Med Inform Decis Mak.

[108] Klack L, Ziefle M, Wilkowska W, Kluge J (2013). Telemedical versus conventional heart patient monitoring: a survey study with German physicians. Int J Technol Assess Health Care.

[109] Kleinpell R, Barden C, Rincon T, McCarthy M, Rufo RJ (2016). Assessing the impact of telemedicine on nursing care in intensive care units. Am J Crit Care.

[110] Koval PG, Kim JJ, Makhlouf T (2018). Pharmacist perception of a mobile application audience response system for remote pharmacy continuing education participants. J Pharm Pract.

[111] Kowitlawakul Y (2011). The technology acceptance model: predicting nurses' intention to use telemedicine technology (eICU). Comput Inform Nurs.

[112] Kuhn E, Eftekhari A, Hoffman JE, Crowley JJ, Ramsey KM, Reger GM, Ruzek JI (2014). Clinician perceptions of using a smartphone app with prolonged exposure therapy. Adm Policy Ment Health.

[113] Kuo KM, Talley PC, Lee C, Yen Y (2015). The influence of telemedicine experience on physicians' perceptions regarding adoption. Telemed J E Health.

[114] Lee SI, Park H, Kim JW, Hwang H, Cho EY, Kim Y, Ha K (2012). Physicians' perceptions and use of a health information exchange: a pilot program in South Korea. Telemed J E Health.

[115] L'Esperance ST, Perry DJ (2016). Assessing advantages and barriers to telemedicine adoption in the practice setting: A MyCareTeam(TM) exemplar. J Am Assoc Nurse Pract.

[116] Liu CF, Cheng TJ (2015). Exploring critical factors influencing physicians' acceptance of mobile electronic medical records based on the dual-factor model: a validation in Taiwan. BMC Med Inform Decis Mak.

[117] Albrecht UV, Afshar K, Illiger K, Becker S, Hartz T, Breil B, Wichelhaus D, von Jan U (2017). Expectancy, usage and acceptance by general practitioners and patients: exploratory results from a study in the German outpatient sector. Digit Health.

[118] Ly BA, Kristjansson E, Labonté R, Bourgeault IL (2018). Determinants of the intention of Senegal's physicians to use telemedicine in their professional activities. Telemed J E Health.

[119] Mairesse GH, Braunschweig F, Klersy K, Cowie MR, Leyva F (2015). Implementation and reimbursement of remote monitoring for cardiac implantable electronic devices in Europe: a survey from the health economics committee of the European Heart Rhythm Association. Europace.

[120] Miller KE, Kuhn E, Owen JE, Taylor K, Yu JS, Weiss BJ, Crowley JJ, Trockel M (2019). Clinician perceptions related to the use of the CBT-I Coach Mobile App. Behav Sleep Med.

[121] Moore MA, Coffman M, Jetty A, Klink K, Petterson S, Bazemore A (2017). Family physicians report considerable interest in, but limited use of, telehealth services. J Am Board Fam Med.

[122] Morilla MD, Sans M, Casasa A, Giménez N (2017). Implementing technology in healthcare: insights from physicians. BMC Med Inform Decis Mak.

[123] Moskowitz A, Chan YY, Bruns J, Levine SR (2010). Emergency physician and stroke specialist beliefs and expectations regarding telestroke. Stroke.

[124] O'Connor S, Andrews T (2018). Smartphones and mobile applications (apps) in clinical nursing education: a student perspective. Nurse Educ Today.

[125] Okazaki S, Castañeda JA, Sanz S, Henseler J (2012). Factors affecting mobile diabetes monitoring adoption among physicians: questionnaire study and path model. J Med Internet Res.

[126] Orruño E, Gagnon MP, Asua J, Abdeljelil AB (2011). Evaluation of teledermatology adoption by health-care professionals using a modified Technology Acceptance Model. J Telemed Telecare.

[127] Putzer GJ, Park Y (2012). Are physicians likely to adopt emerging mobile technologies? Attitudes and innovation factors affecting smartphone use in the Southeastern United States. Perspect Health Inf Manag.

[128] El Amrani L, Engberink AO, Ninot G, Hayot M, Carbonnel F (2017). Connected health devices for health care in French general medicine practice: cross-sectional study. JMIR Mhealth Uhealth.

[129] Rho MJ, Choi IY, Lee J (2014). Predictive factors of telemedicine service acceptance and behavioral intention of physicians. Int J Med Inform.

[130] Rogove HJ, McArthur D, Demaerschalk BM, Vespa PM (2012). Barriers to telemedicine: survey of current users in acute care units. Telemed J E Health.

[131] Sadoughi F, Erfannia L, Sancholi M, Salmani F, Sarsarshahi A (2017). Teleradiology in Southeast Iran: Evaluating the Views of Senior Executives and Radiologists. Health Care Manag (Frederick).

[132] Saigi-Rubió F, Jiménez-Zarco A, Torrent-Sellens J (2016). Determinants of the intention to use telemedicine: evidence from primary care physicians. Int J Technol Assess Health Care.

[133] Saigí-Rubió F, Torrent-Sellens J, Jiménez-Zarco A (2014). Drivers of telemedicine use: comparative evidence from samples of Spanish, Colombian and Bolivian physicians. Implement Sci.

[134] Sandholzer M, Deutsch T, Frese T, Winter A (2015). Predictors of students' self-reported adoption of a smartphone application for medical education in general practice. BMC Med Educ.

[135] Schmeer R, Behrends M, Kupka T, Meyenburg-Altwarg I, Marschollek M (2016). Use and acceptance of mobile technology by hospital nurses in Germany. Stud Health Technol Inform.

[136] Sezgin E, Özkan-Yildirim S, Yildirim S (2018). Understanding the perception towards using mHealth applications in practice. Inf Dev.

[137] Sims MH, Fagnano M, Halterman JS, Halterman MW (2016). Provider impressions of the use of a mobile crowdsourcing app in medical practice. Health Informatics J.

[138] Smith PB, Buzi RS (2014). Reproductive health professionals' adoption of emerging technologies for health promotion. Health Informatics J.

[139] Asua J, Orruño E, Reviriego E, Gagnon MP (2012). Healthcare professional acceptance of telemonitoring for chronic care patients in primary care. BMC Med Inform Decis Mak.

[140] Steinschaden T, Petersson G, Astrand B (2009). Physicians' attitudes towards eprescribing: a comparative web survey in Austria and Sweden. Inform Prim Care.

[141] Uscher-Pines L, Kahn JM (2014). Barriers and facilitators to pediatric emergency telemedicine in the United States. Telemed J E Health.

[142] van Houwelingen CT, Barakat A, Best R, Boot WR, Charness N, Kort HS (2015). Dutch nurses' willingness to use home telehealth: implications for practice and education. J Gerontol Nurs.

[143] Villalba-Mora E, Casas I, Lupiañez-Villanueva F, Maghiros I (2015). Adoption of health information technologies by physicians for clinical practice: the Andalusian case. Int J Med Inform.

[144] Yaman H, Yavuz E, Er A, Vural R, Albayrak Y, Yardimci A, Asilkan O (2016). The use of mobile smart devices and medical apps in the family practice setting. J Eval Clin Pract.

[145] Zailani S, Gilani MS, Nikbin D, Iranmanesh M (2014). Determinants of telemedicine acceptance in selected public hospitals in Malaysia: clinical perspective. J Med Syst.

[146] Zhang H, Cocosila M, Archer N (2010). Factors of adoption of mobile information technology by homecare nurses: a technology acceptance model 2 approach. Comput Inform Nurs.

[147] Ayatollahi H, Mirani N, Nazari F, Razavi N (2018). Iranian healthcare professionals' perspectives about factors influencing the use of telemedicine in diabetes management. World J Diabetes.

[148] Brieux HM, Benitez S, Otero C, Luna D, Masud JH, Marcelo A, Househ M, Hullin C, Villalba C, Indarte S, Guillen S, Otero P, Campos F, Baum A, de Quirós FG (2017). Cultural problems associated with the implementation of eHealth. Stud Health Technol Inform.

[149] Dünnebeil S, Sunyaev A, Blohm I, Leimeister JM, Krcmar H (2012). Determinants of physicians' technology acceptance for e-health in ambulatory care. Int J Med Inform.

[150] Duhm J, Fleischmann R, Schmidt S, Hupperts H, Brandt SA (2016). Mobile electronic medical records promote workflow: physicians' perspective from a survey. JMIR Mhealth Uhealth.

[151] Alajlani M, Clarke M (2013). Effect of culture on acceptance of telemedicine in Middle Eastern countries: case study of Jordan and Syria. Telemed J E Health.

[152] Ariens LF, Schussler-Raymakers FM, Frima C, Flinterman A, Hamminga E, Arents BW, Bruijnzeel-Koomen CA, de Bruin-Weller MS, van Os-Medendorp H (2017). Barriers and facilitators to eHealth use in daily practice: perspectives of patients and professionals in dermatology. J Med Internet Res.

[153] Iacono T, Dissanayake C, Trembath D, Hudry K, Erickson S, Spong J (2016). Family and practitioner perspectives on telehealth for services to young children with autism. Stud Health Technol Inform.

[154] Jamu JT, Lowi-Jones H, Mitchell C (2016). Just in time? Using QR codes for multi-professional learning in clinical practice. Nurse Educ Pract.

[155] Jeon J, Taneva S, Kukreti V, Trbovich P, Easty AC, Rossos PG, Cafazzo JA (2014). Toward successful migration to computerized physician order entry for chemotherapy. Curr Oncol.

[156] Loh P, Flicker L, Horner B (2009). Attitudes toward information and communication technology (ICT) in residential aged care in Western Australia. J Am Med Dir Assoc.

[157] Lapão LV, da Silva MM, Gregório J (2017). Implementing an online pharmaceutical service using design science research. BMC Med Inform Decis Mak.

[158] Lygidakis C, Wallace P, Tersar C, Marcatto F, Ferrante D, Vedova RD, Scafuri F, Scafato E, Struzzo P (2016). Download Your Doctor: implementation of a digitally mediated personal physician presence to enhance patient engagement with a health-promoting internet application. JMIR Res Protoc.

[159] Mueller KJ, Potter AJ, MacKinney AC, Ward MM (2014). Lessons from tele-emergency: improving care quality and health outcomes by expanding support for rural care systems. Health Aff (Millwood).

[160] Muigg D, Kastner P, Modre-Osprian R, Haluza D, Duftschmid G (2018). Is Austria ready for telemonitoring? A readiness assessment among doctors and patients in the field of diabetes. Stud Health Technol Inform.

[161] Nerminathan A, Harrison A, Phelps M, Alexander S, Scott KM (2017). Doctors' use of mobile devices in the clinical setting: a mixed methods study. Intern Med J.

[162] Odnoletkova I, Buysse H, Nobels F, Goderis G, Aertgeerts B, Annemans L, Ramaekers D (2016). Patient and provider acceptance of telecoaching in type 2 diabetes: a mixed-method study embedded in a randomised clinical trial. BMC Med Inform Decis Mak.

[163] Bailey JV, Tomlinson N, Hobbs LJ, Webster R (2017). Challenges and opportunities in evaluating a digital sexual health intervention in a clinic setting: Staff and patient views. Digit Health.

[164] Orchard J, Lowres N, Freedman SB, Ladak L, Lee W, Zwar N, Peiris D, Kamaladasa Y, Li J, Neubeck L (2016). Screening for atrial fibrillation during influenza vaccinations by primary care nurses using a smartphone electrocardiograph (iECG): A feasibility study. Eur J Prev Cardiol.

[165] Payne KF, Weeks L, Dunning P (2014). A mixed methods pilot study to investigate the impact of a hospital-specific iPhone application (iTreat) within a British junior doctor cohort. Health Informatics J.

[166] Possemato K, Kuhn E, Johnson EM, Hoffman JE, Brooks E (2017). Development and refinement of a clinician intervention to facilitate primary care patient use of the PTSD Coach app. Transl Behav Med.

[167] Quanbeck A, Gustafson DH, Marsch LA, Chih M, Kornfield R, McTavish F, Johnson R, Brown RT, Mares M, Shah DV (2018). Implementing a mobile health system to integrate the treatment of addiction into primary care: a hybrid implementation-effectiveness study. J Med Internet Res.

[168] Ray KN, Felmet KA, Hamilton MF, Kuza CC, Saladino RA, Schultz BR, Watson RS, Kahn JM (2017). Clinician attitudes toward adoption of pediatric emergency telemedicine in rural hospitals. Pediatr Emerg Care.

[169] Shaw RJ, Kaufman MA, Bosworth HB, Weiner BJ, Zullig LL, Lee SD, Kravetz JD, Rakley SM, Roumie CL, Bowen ME, Del Monte PS, Oddone EZ, Jackson GL (2013). Organizational factors associated with readiness to implement and translate a primary care based telemedicine behavioral program to improve blood pressure control: the HTN-IMPROVE study. Implement Sci.

[170] Tahamtan I, Pajouhanfar S, Sedghi S, Azad M, Roudbari M (2017). Factors affecting smartphone adoption for accessing information in medical settings. Health Info Libr J.

[171] Taylor A, Wade V, Morris G, Pech J, Rechter S, Kidd M, Carati C (2016). Technology support to a telehealth in the home service: qualitative observations. J Telemed Telecare.

[172] Walker L, Clendon J (2016). The case for end-user involvement in design of health technologies. J Telemed Telecare.

[173] Williamson KM, Muckle J (2018). Students' perception of technology use in nursing education. Comput Inform Nurs.

[174] Bidmead E, Marshall A (2016). A case study of stakeholder perceptions of patient held records: the Patients Know Best (PKB) solution. Digit Health.

[175] Zhang Y, Koch S (2015). Mobile health apps in Sweden: what do physicians recommend?. Stud Health Technol Inform.

[176] Bramley G, Mangan C, Conroy M (2019). Using telemonitoring to support personal care planning for adults with learning disabilities. J Telemed Telecare.

[177] Brown W, Giguere R, Sheinfil A, Ibitoye M, Balan I, Ho T, Brown B, Quispe L, Sukwicha W, Lama JR, Carballo-Diéguez A, Cranston RD (2018). Challenges and solutions implementing an SMS text message-based survey CASI and adherence reminders in an international biomedical HIV PrEP study (MTN 017). J Biomed Inform.

[178] Chang F, Paramsothy T, Roche M, Gupta NS (2017). Patient, staff, and clinician perspectives on implementing electronic communications in an interdisciplinary rural family health practice. Prim Health Care Res Dev.

[179] Charani E, Kyratsis Y, Lawson W, Wickens H, Brannigan ET, Moore LS, Holmes AH (2013). An analysis of the development and implementation of a smartphone application for the delivery of antimicrobial prescribing policy: lessons learnt. J Antimicrob Chemother.

[180] Cox A, Illsley M, Knibb W, Lucas C, O'Driscoll M, Potter C, Flowerday A, Faithfull S (2011). The acceptability of e-technology to monitor and assess patient symptoms following palliative radiotherapy for lung cancer. Palliat Med.

[181] Ehrler F, Ducloux P, Wu DT, Lovis C, Blondon K (2018). Acceptance of a mobile application supporting nurses workflow at patient bedside: results from a pilot study. Stud Health Technol Inform.

[182] Radhakrishnan K, Xie B, Berkley A, Kim M (2016). Barriers and facilitators for sustainability of tele-homecare programs: a systematic review. Health Serv Res.

[183] Davis MM, Freeman M, Kaye J, Vuckovic N, Buckley DI (2014). A systematic review of clinician and staff views on the acceptability of incorporating remote monitoring technology into primary care. Telemed J E Health.

[184] Hickson R, Talbert J, Thornbury WC, Perin NR, Goodin AJ (2015). Online medical care: the current state of 'eVisits' in acute primary care delivery. Telemed J E Health.

[185] Koivunen M, Saranto K (2018). Nursing professionals' experiences of the facilitators and barriers to the use of telehealth applications: a systematic review of qualitative studies. Scand J Caring Sci.

[186] Kumar S, Merchant S, Reynolds R (2013). Tele-ICU: efficacy and cost-effectiveness approach of remotely managing the critical care. Open Med Inform J.

[187] Lewis ER, Thomas CA, Wilson ML, Mbarika VW (2012). Telemedicine in acute-phase injury management: a review of practice and advancements. Telemed J E Health.

[188] Li L, Cotton A (2019). A systematic review of nurses' perspectives toward the telemedicine intensive care unit: a basis for supporting its future implementation in China?. Telemed J E Health.

[189] Penny RA, Bradford NK, Langbecker D (2018). Registered nurse and midwife experiences of using videoconferencing in practice: a systematic review of qualitative studies. J Clin Nurs.

[190] Daniel F, Jabak S, Sasso R, Chamoun Y, Tamim H (2018). Patient-physician communication in the era of mobile phones and social media apps: cross-sectional observational study on Lebanese physicians' perceptions and attitudes. JMIR Med Inform.

[191] Jungwirth D, Haluza D (2019). Information and communication technology and the future of healthcare: results of a multi-scenario Delphi survey. Health Informatics J.

[192] Ahmad F, Norman C, O'Campo P (2012). What is needed to implement a computer-assisted health risk assessment tool? An exploratory concept mapping study. BMC Med Inform Decis Mak.

[193] Mishori R, Anastario M, Naimer K, Varanasi S, Ferdowsian H, Abel D, Chugh K (2017). mJustice: preliminary development of a mobile app for medical-forensic documentation of sexual violence in low-resource environments and conflict zones. Glob Health Sci Pract.

[194] Cunningham DL, Connors EH, Lever N, Stephan SH (2013). Providers' perspectives: utilizing telepsychiatry in schools. Telemed J E Health.

[195] Bishop TF, Press MJ, Mendelsohn JL, Casalino LP (2013). Electronic communication improves access, but barriers to its widespread adoption remain. Health Aff (Millwood).

[196] Putzer GJ, Park Y (2010). The effects of innovation factors on smartphone adoption among nurses in community hospitals. Perspect Health Inf Manag.

[197] Voncken-Brewster V, Tange H, Moser A, Nagykaldi Z, de Vries H, van der Weijden T (2014). Integrating a tailored e-health self-management application for chronic obstructive pulmonary disease patients into primary care: a pilot study. BMC Fam Pract.

[198] Murray J (2017). Oxford Dictionary Of English.

[199] Oudshoorn N, Rommes E, Stienstra M (2004). Configuring the user as everybody: gender and design cultures in information and communication technologies. Sci Technol Hum Values.

