# Correction: Social, Organizational, and Technological Factors Impacting Clinicians’ Adoption of Mobile Health Tools: Systematic Literature Review

**DOI:** 10.2196/37747

**Published:** 2022-03-10

**Authors:** Christine Jacob, Antonio Sanchez-Vazquez, Chris Ivory

**Affiliations:** 1 Anglia Ruskin University Cambridge United Kingdom; 2 University of Applied Sciences Northwestern Switzerland Brugg Switzerland; 3 Innovation and Management Practice Research Centre, Anglia Ruskin University Cambridge United Kingdom

In “Social, Organizational, and Technological Factors Impacting Clinicians’ Adoption of Mobile Health Tools: Systematic Literature Review” (JMIR Mhealth Uhealth 2020;8(2):e15935), one error was noted.

In the originally published manuscript, Table 4 was incorrectly displayed as an identical copy of Table 3. The complete, corrected version of Table 4 has now been included in the corrected version of the manuscript.

The full corrected table is included below.

The correction will appear in the online version of the paper on the JMIR Publications website on March 10, 2022, together with the publication of this correction notice. Because this was made after submission to PubMed, PubMed Central, and other full-text repositories, the corrected article has also been resubmitted to those repositories.

**Table 4:** Patient-related factors and their occurrence, with references.

Factor	Subthemes	References
Quality and efficiency of care (n=77)	Examples: treatment outcomes, clinical delivery, patient monitoring, and treatment compliance	[1-3,5,9,10,34,36-40,42-44,47-49,52-57,61-64,66,69,72,75,78,80,83,86,88,89,92,93, 100,103,107-109,114,115,120,122,125,127,130,131,137,140,146,157,159, 160,162,165,166,168,169,174,175,180,182-186,188,190,194,196]
Provider-patient communication (n=53)	Quality and ease of communication between patients and the care team	[3,5,9,10,34,36,41,43,46,47,49,50,52,55,58,59,66,67,72,73,75,77,78,80-82, 84-86,88-91,103,115,122,127,128,150,156,160,163,172,174,175,180,182,185,189, 190,194,195]
Access to care (n=41)	Enhancing patients’ access to care and reaching the underserved	[1-5,8,34,39,43,47,54,55,60,62,64,67,73,75,78,81,82,91,93,96,99,102, 123,129,130,156,159,162,169,174,178,180,182,184,187,189,190, 194,195]
Patient consent, comfort, and preference (n=30)	Comfort with technology, personal preferences, and the ease of getting an informed consent from the patients	[1-3,9,39,43,49,52,53,60,61,66,79,86,90,104,122,123,132,153,163, 174,176,180,184,185,191,192,194,195]
Applicability and appropriateness (n=22)	The suitability of patients on the basis of their needs and characteristics	[9,41,57,61,62,66,68,71,73,75,79,81,86,88-91,103,104,108,162,182,185,189]
Empowerment and engagement (n=21)	Opportunity to empower and reassure patients and increase their engagement in managing their condition	[5,34,41,48,62,70,71,73,77,78,88,89,115,120,128,157,162,166,175,180,190]
Safety (n=19)	Patient safety and the safety of clinical practice	[10,44,63,66,72,78,81,86,103,109,140,147,155,176,179,188-190,195]
Digital divide (n=15)	Age, living standard, and access to technology	[49,53,55,60,62,73,75,79,81,84,120,123,138,185,191]
Education (n=12)	Better patient education and awareness	[52,53,60,75,81,86,88,162,164,168,183,190]
Service abuse, overreliance (n=8)	Patient overdependence on practitioner support	[3,41,62,77,78,156,169,182]
Data and surveillance–related anxiety (n=6)	Worries and anxiety related to the understanding and interpretation of data, or the feeling of being observed	[46,62,78,128,140,190]
Sustainability (n=3)	Long-term commitment and use	[5,167,169]
Gate keeping by clinicians (n=2)	Protective or paternalistic attitudes of the care team	[97,180]

