# Supplementary figures and images for: Social, Organizational, and Technological Factors Impacting Clinicians’ Adoption of Mobile Health Tools: Systematic Literature Review
Source: JMIR Mhealth Uhealth. 2020 Feb 20;8(2):e15935. doi: 10.2196/15935 (PMC7059085; doi:10.2196/15935)

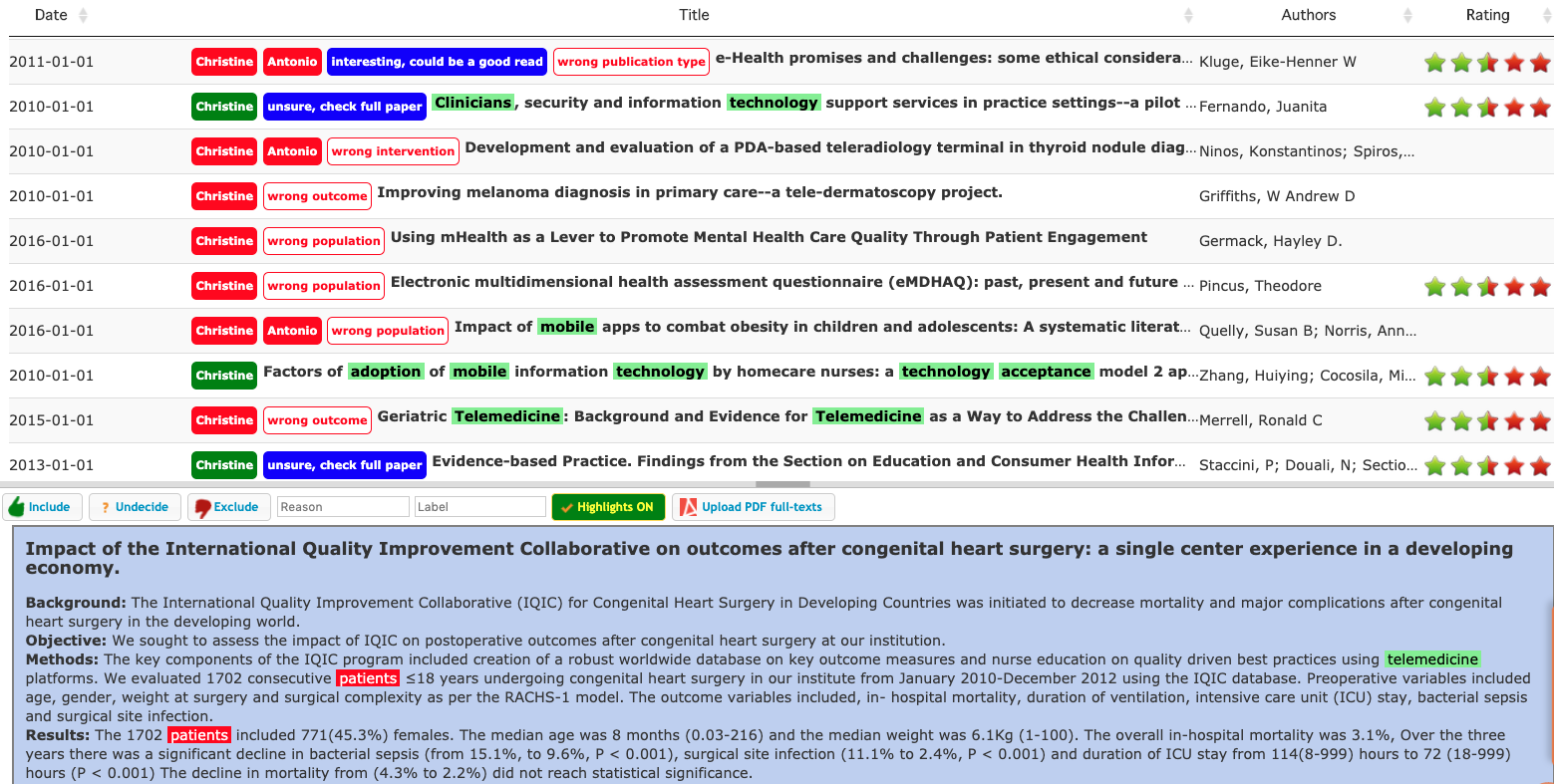

Supplement: Multimedia Appendix 1 [file mhealth_v8i2e15935_app1.png]
